# Evolutionary activation of acidic chitinase in herbivores through the H128R mutation in ruminant livestock

**DOI:** 10.1016/j.isci.2023.107254

**Published:** 2023-07-03

**Authors:** Eri Tabata, Ikuto Kobayashi, Takuya Morikawa, Akinori Kashimura, Peter O. Bauer, Fumitaka Oyama

**Affiliations:** 1 Department of Chemistry and Life Science, Kogakuin University, Hachioji, Tokyo 192-0015, Japan; 2 Research Fellow of Japan Society for the Promotion of Science (PD), Koujimachi, Chiyoda-ku, Tokyo 102-0083, Japan; 3 Bioinova a.s., Videnska 1083, 142 00 Prague, Czech Republic

**Keywords:** Evolutionary biology, Molecular biology, Zoology

## Abstract

Placental mammals' ancestors were insectivores, suggesting that modern mammals may have inherited the ability to digest insects. Acidic chitinase (Chia) is a crucial enzyme hydrolyzing significant component of insects' exoskeleton in many species. On the other hand, herbivorous animal groups, such as cattle, have extremely low chitinase activity compared to omnivorous species, e.g., mice. The low activity of cattle Chia has been attributed to R128H mutation. The presence of either of these amino acids correlates with the feeding behavior of different bovid species with R and H determining the high and low enzymatic activity, respectively. Evolutionary analysis indicated that selective constraints were relaxed in 67 herbivorous *Chia* in Cetartiodactyla. Despite searching for another Chia paralog that could compensate for the reduced chitinase activity, no active paralogs were found in this order. Herbivorous animals' *Chia* underwent genetic alterations and evolved into a molecule with low activity due to the chitin-free diet.

## Highlights

• Herbivorous cattle have low chitinase activity due to R128H mutation
• Insect-eating bovids have high chitinase activity due to the retention of R128
• No active Chia paralogs were found in herbivorous Cetartiodactyla species
• Selective constraints were relaxed in 67 herbivorous *Chia* genes in Cetartiodactyla

## Graphical abstract

**Figure:**
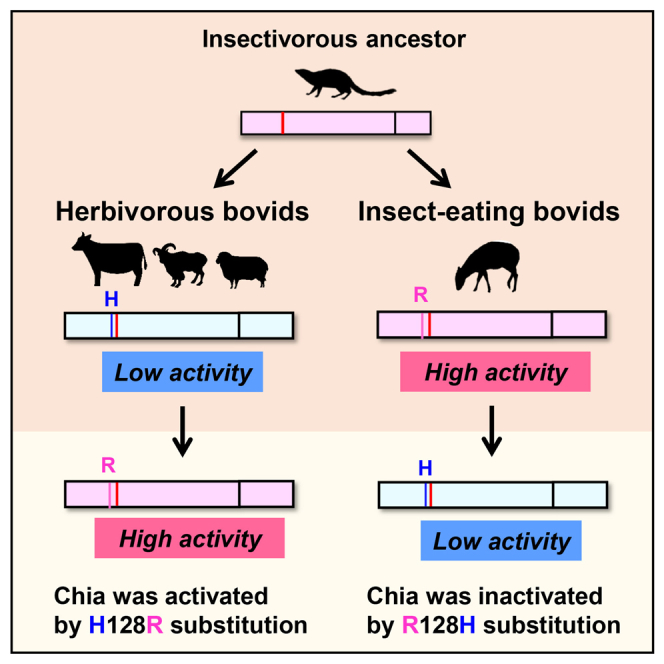

## Introduction

Genomics and fossil records suggest that the ancestors of placental mammals mainly consumed insects.^1^ Therefore, extant species may have inherited a gastrointestinal system able to digest chitin abundantly in insects.

Chitin is a linear polymer of β-1, 4-linked *N*-acetyl-D-glucosamine (GlcNAc). It is the second most abundant polysaccharide on earth and is a major structural component in many organisms, such as insects, crustaceans, nematodes, and fungi.^2^^,^^3^ Chitinases hydrolyze the chitin’s β-1, 4 glycoside bonds.^3^^,^^4^^,^^5^ While mammals do not synthesize chitin, they possess two functional chitinases, active in mice and humans. Chitotriosidase (Chit1) was first identified in Gaucher disease patients.^6^^,^^7^^,^^8^ Acidic chitinase (Chia; also reported as acidic mammalian chitinase, AMCase) was discovered later and was named for its acidic isoelectric point.^9^

Since Chia expression is significantly altered under several pathological conditions, such as asthma and allergic inflammation,^10^^,^^11^^,^^12^^,^^13^^,^^14^^,^^15^^,^^16^ it has attracted considerable scientific attention. Some polymorphisms and haplotypes in the Chia gene are associated with human bronchial asthma.^17^^,^^18^^,^^19^ In addition, Chia was shown to be a constitutively produced enzyme essential for the degradation of environmentally derived chitin in the airways to maintain lung functions.^20^^,^^21^

Chia is an enzyme that digests insect chitin in the stomachs of insectivorous and omnivorous animals, such as bats, mice, chickens, pigs, pangolins, common marmoset, and crab-eating monkeys.^22^^,^^23^^,^^24^^,^^25^^,^^26^^,^^27^^,^^28^^,^^29^^,^^30^^,^^31^ However, in some animals, such as dogs (carnivores) and cattle (herbivores), Chia expression and activity are very low, resulting in impaired chitin digestion.^32^ These findings suggest that dietary habits influence Chia expression and activity and determine chitin digestibility in different animals.^32^

Genetic studies have revealed that insect-eating placental mammals' predecessors carry five *Chia* paralogs. However, these genes have been lost multiple times in mammals whose diet comprises only limited amounts of chitin-containing organisms.^33^ It has been shown that mice, dogs, and cattle possess only one *Chia* paralog, *Chia5*.^33^ Additionally, several *Chia* paralogous genes have been found in nonhuman primates linked to insect consumption and body size.^34^

We recently reported that replacing F214L with A216G led to dog Chia activation and showed that a non-insect-based diet had caused structural and functional changes during evolution in Carnivora.^35^ However, the cause of Chia’s activity reduction in herbivorous species, including cattle, has not been elucidated.

Ruminants are important terrestrial herbivores and include at least 200 extant domestic and wild species. Cattle, goats, and sheep are one of the most important livestock in the world, producing milk, meat, and leather. Due to their economic importance, the whole genome sequences of the above species were revealed in 2009–2014.^36^^,^^37^^,^^38^

This report shows that single amino acid substitution can activate cattle Chia by introducing H128R. Shift to herbivorous diets caused relaxation of the *Chia* functional constraints and suppressed its enzymatic activity. Our results reveal genetic changes associated with dietary specialization and aid in understanding herbivorous evolution.

## Results

### Exon 5 is involved in reducing the cattle Chia activity

**Figure 1:**
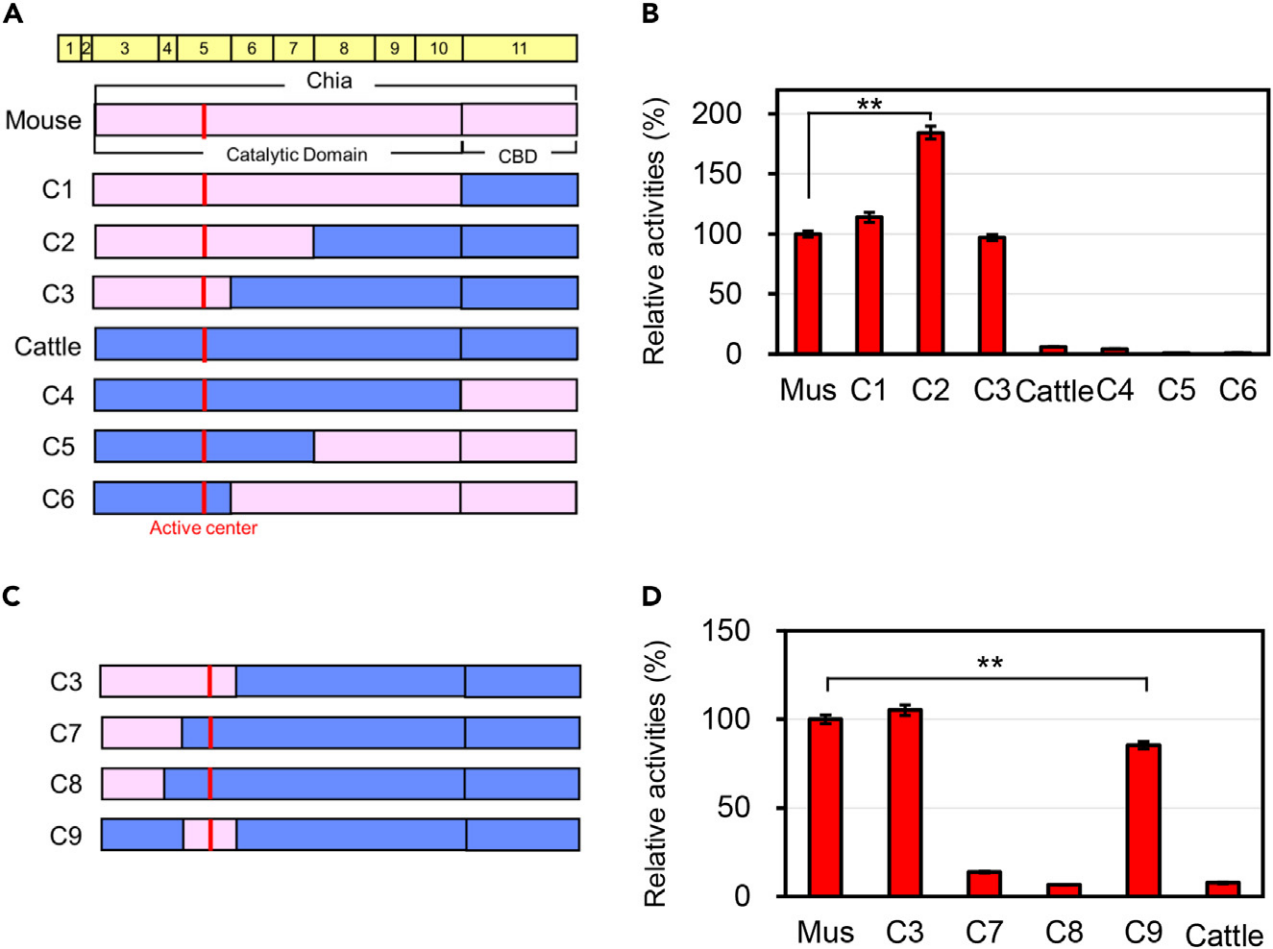
Exon 5 is responsible for the low activity of cattle Chia (A) Schematic representation of *E. coli*-expressed Chia chimeric proteins. The amino acid sequences are color-coded: pink, mouse sequence; blue, cattle sequence. (B) Comparison of the chitinolytic activities of Chia proteins outlined in panel A. Error bars represent mean ± SD from a single experiment conducted in triplicate. Welch’s t-test compared data. ^∗∗^p < 0.01. p values were determined using Welch’s t-test. (C) Schematic representation of *E. coli*-expressed Chia chimeric proteins. (D) Comparison of the chitinolytic activities of chimeric proteins outlined in panel C with mouse and cattle Chia.

Cattle Chia has significantly lower chitinase activity than the mouse enzyme.^32^ To identify the regions responsible for such reduced activity, we constructed and expressed chimeric mouse-cattle enzymes in *Escherichia coli* (*E. coli*) (Figures 1A and S1–S4). The chitinolytic activity of each chimera was measured using a synthetic fluorogenic substrate 4-methyl umbelliferyl β-D-*N*, *N*′-diacetyl chitobioside [4-MU-(GlcNAc)_2_].

Chimeras C1 (coded by mouse exons 3–10 and cattle exon 11) and C3 (mouse exons 3–5 and cattle exons 6–11) exhibited chitinolytic activity comparable to that of wild-type (WT) mouse Chia (Figures 1B and S2–S4). Chimera C2 (mouse exons 3–7 and cattle exons 8–11) showed significant activation (almost twice as much as the mouse enzyme), likely due to the synergistic effect of amino acids from mouse and cattle (Figures 1B and S2–S4). However, the chitinolytic activities were very low in chimeras C4 (cattle exons 3–10 and mouse exon 11), C5 (cattle exons 3–7 and mouse exons 8–11), and C6 (cattle exons 3–5 and mouse exons 6–11), similarly to the cattle enzyme (Figures 1B and S2–S4). These results indicate that exons 3–5 are crucial in chitinolytic activity in Chia, with mouse sequence activating and cattle sequence deactivating the enzyme.

To further investigate the crucial area, we constructed coded by mouse exons 3–4 and cattle exons 5–11 (C7) and by mouse exon 3 and cattle exons 4–11 (C8) (Figures 1C, S4, and S5). Both chimeras displayed significantly lower activity than C3 (containing mouse exon 5) (Figure 1D), suggesting that exon 5 represents the critical region in the cattle Chia activation. Therefore, we exchanged exon 5 in cattle Chia by the mouse sequence (chimera C9) (Figures 1C, S4, and S5), causing restoration of about 80% of the mouse enzyme activity, suggesting that exon 5 is involved in the low activity of cattle Chia (Figure 1D).

### H128R activated the chitinolytic activity of cattle Chia

**Figure 2:**
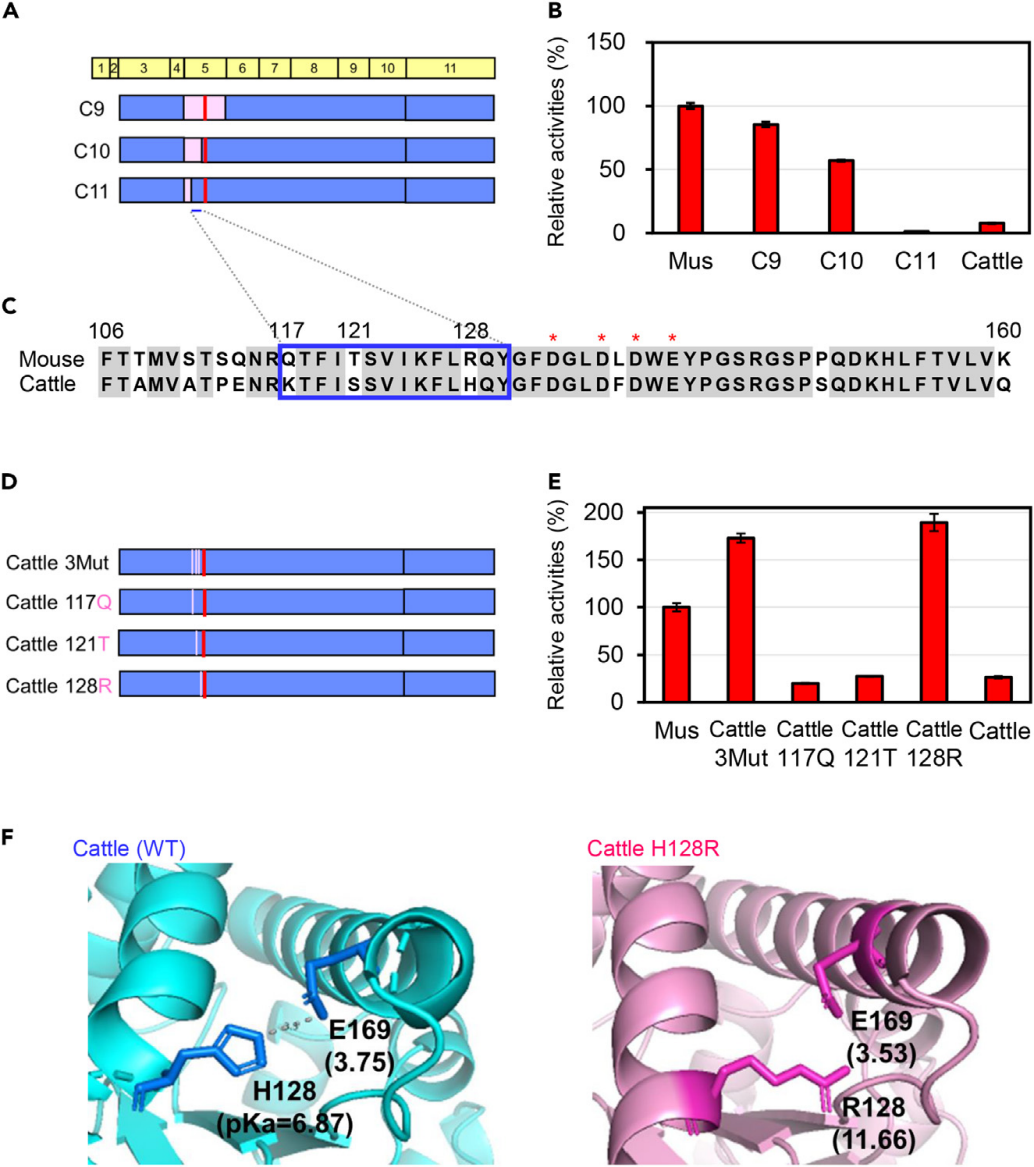
Narrowing down the region and identifying amino acids that affect the cattle Chia activity (A) Schematic representation of *E. coli*-expressed Chia chimeric proteins. The amino acid sequences are color-coded: pink, mouse sequence; blue, cattle sequence. (B) Comparison of the chitinolytic activities of mutant proteins with mouse and cattle Chia. Error bars represent mean ± SD from a single experiment conducted in triplicate. (C) Amino acid sequences of exon 5 regions of mouse and cattle Chia. The amino acids that differ between the enzymes are colored: pink, mouse sequence; blue, cattle sequence. The blue box highlights the region involved in the activity reduction. Asterisks indicate the conserved motif for the chitinase catalytic site (DXXDXDXE). (D) Schematic representation of *E. coli*-expressed Chia mutant proteins. (E) Comparison of the chitinolytic activities of mutant proteins outlined in panel E with mouse and cattle Chia. (F) A homology model of cattle Chia’s wild-type (blue) and the mutant (pink) proteins.

Ten residues are different in exon 5 between both enzymes (Figure 2C). Therefore, we constructed chimeras C10 and C11 that introduced a part of the mouse exon 5 regions to the cattle enzyme (Figures 2A, S4, and S5). The activity of the chimera (C11) with the cattle sequence in the middle region of exon 5 was significantly reduced (Figure 2B). These results strongly suggest that residues at positions 117, 121, and 128 are involved in the low activity of cattle Chia (Figure 2C).

To identify the amino acids involved in this activation, we introduced K117Q, S121T, and H128R to cattle Chia (Figures 2D, S4, and S6). The mutant Chia protein carrying 3 amino acid mutation (3Mut) showed higher activity than the WT mouse enzyme, indicating that these amino acids were indeed involved in Chia’s low activity (Figure 2E).

To narrow down the amino acids involved in this activation, we introduced single amino acid substitution to cattle Chia (Cattle117Q, Cattle121T, and Cattle128R mutants) (Figures 2D, S4, and S6). The mutant (MT) proteins carrying K117Q and S121T showed no enzyme activation. However, the H128R mutant achieved a ∼10-fold activity increase (Figure 2E). These results indicate that histidine at position 128 is the cause of low activity in cattle Chia.

AlphaFold2, an artificial intelligence program,^39^^,^^40^ predicted the WT and MT cattle Chia protein structures. By calculating the pKa of the protein,^41^ H128 (pKa = 6.87) was located on the α-helix and interacted with E169 on another α-helix (Figure 2F, left). Chia has a catalytic domain (CatD) consisting of the triose phosphate isomerase (TIM barrel) fold, which involves the groove of the substrate bond by essential tunneling. For mutant enzymes, R128 (pKa = 11.66) does not interact with E169 (Figure 2F, right). Therefore, this amino acid substitution at position 128 may alter the interaction between the two α-helices of the TIM barrel, affecting the overall structure and catalytic function.

### A dietary habit of the bovids determines the amino acid at position 128 in Chia

**Figure 3:**
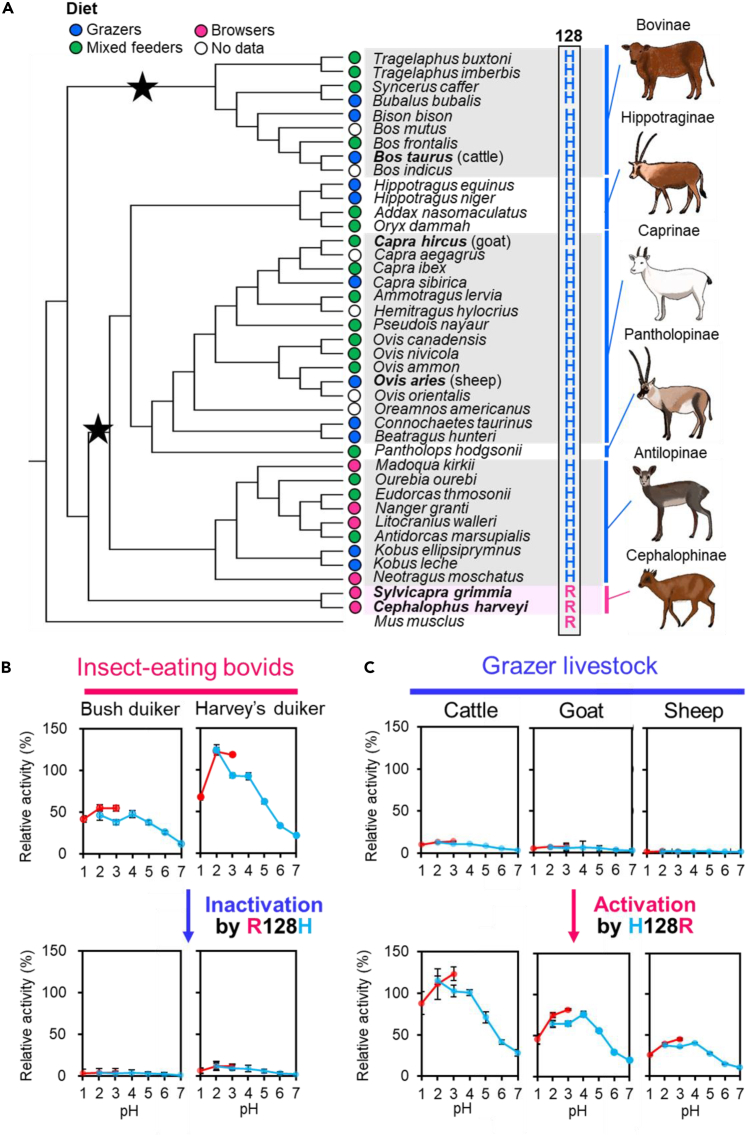
Herbivorous livestock has histidine, while the insect-eating species have arginine at position 128 (A) Phylogenetic tree of 41 Bovidae species. Phylogenetic relationships and divergence times were obtained from TimeTree v3.0 (http://www.timetree.org/). Stars indicate an inactivating event, and the timing is unknown. Each species' main diet is shown in the closed circle. The amino acids located at 128, which are involved in the low activity of cattle Chia, are shown. The illustrations were drawn by Eri Tabata. (B) Comparing bush and Harvey’s duikers' chitinolytic activities of WT or MT Chia proteins. Error bars represent mean ± SD from a single experiment conducted in triplicate. (C) Comparing the chitinolytic activities of cattle, goat, and sheep WT or MT Chia proteins. The relative activity when the mouse Chia activity level at pH 2.0 was set to 100% is shown. Error bars represent mean ± SD from a single experiment conducted in triplicate.

Bovidae is the broadest family in Ruminantia, comprising 143 species, including several domesticated species (cattle, goat, and sheep).^42^ To estimate the timing of the low activity of cattle Chia, we analyzed the completeness of 41 bovid Chia Open Reading Frames (ORFs). The *Chia* nucleotide sequences of these bovids were obtained from the NCBI Genome database (https://www.ncbi.nlm.nih.gov/genome/; Tables S1 and S2; Data S1, available in the supplemental information at https://doi.org/10.1016/j.isci.2023.107254). A phylogenetic tree was obtained from TimeTree (Figure 3A).

Ruminant species can be divided into three feeding behaviors: browsing (dicotyledonous plants including leaves, stems, bark, fruits, etc.), grazing (grass), and mixed feeding (both browsing and grazing).^43^ Based on the previous study,^44^ we classified each of the 34 bovids (Figure 3A).

Most bovids conserved H128, suggesting that the low activity of the Chia event occurred in a common ancestor of these lineages. However, some species (bush and Harvey’s duiker) retained R128 (Figure 3A, lower). Therefore, referring to this phylogenetic tree, it is possible that Bovidae’s Chia activity reduction event occurred multiple times (Figure 3A).

### H128R mutation can activate ruminant livestock Chia

Duiker is found primarily in wooded rather than grassy areas, feeds on leaves, buds, seeds, and fruits, and often consumes small animals and insects. As shown in Figure 3A, bush and Harvey’s duiker Chia retained R128, suggesting they exhibit high chitinolytic activity. We confirmed this assumption by evaluating recombinant bush and Harvey’s duiker Chia and observed 5 and 12 times higher activity than cattle Chia (Figure 3B, upper; Figures S7 and S8). The introduction of the R128H mutation inactivated both Chia enzymes, confirming the importance of this residue for the chitinase activity (Figure 3B, lower; Figures S7 and S8).

Goats and sheep belong to different groups of Bovidae. Cattle and sheep prefer grass, while goats feed on a wider range of plants (Figure 3A). Similarly to cattle, goats, and sheep Chia showed very low levels of chitinase activity (Figure 3C, upper; Figures S8 and S9), further corroborating the role of H128.

Next, we attempted to activate these Chia proteins introducing the H128R mutation. This substitution led to a 10-fold increase of the activity in all enzymes at pH 2.0–4.0 reaching (Figure 3C, lower; Figures S8 and S9), indicating that H128R mutation can activate ruminant livestock Chia.

### The selective constraints were relaxed in ruminants *Chia* in Cetartiodactyla

Cetartiodactyla (Artiodactyla and Cetacea) is one of the most diverse orders of mammals. It includes artiodactyls (cattle, deer, giraffes, pigs, hippos, and camels) and cetaceans (whales and dolphins). Within these species, there are extensive variations in morphology and habitat. Most are herbivores, pig and peccary are omnivores, and whales are carnivores, feeding on chitin-containing organisms such as shrimp, squid, and krill.

**Figure 4:**
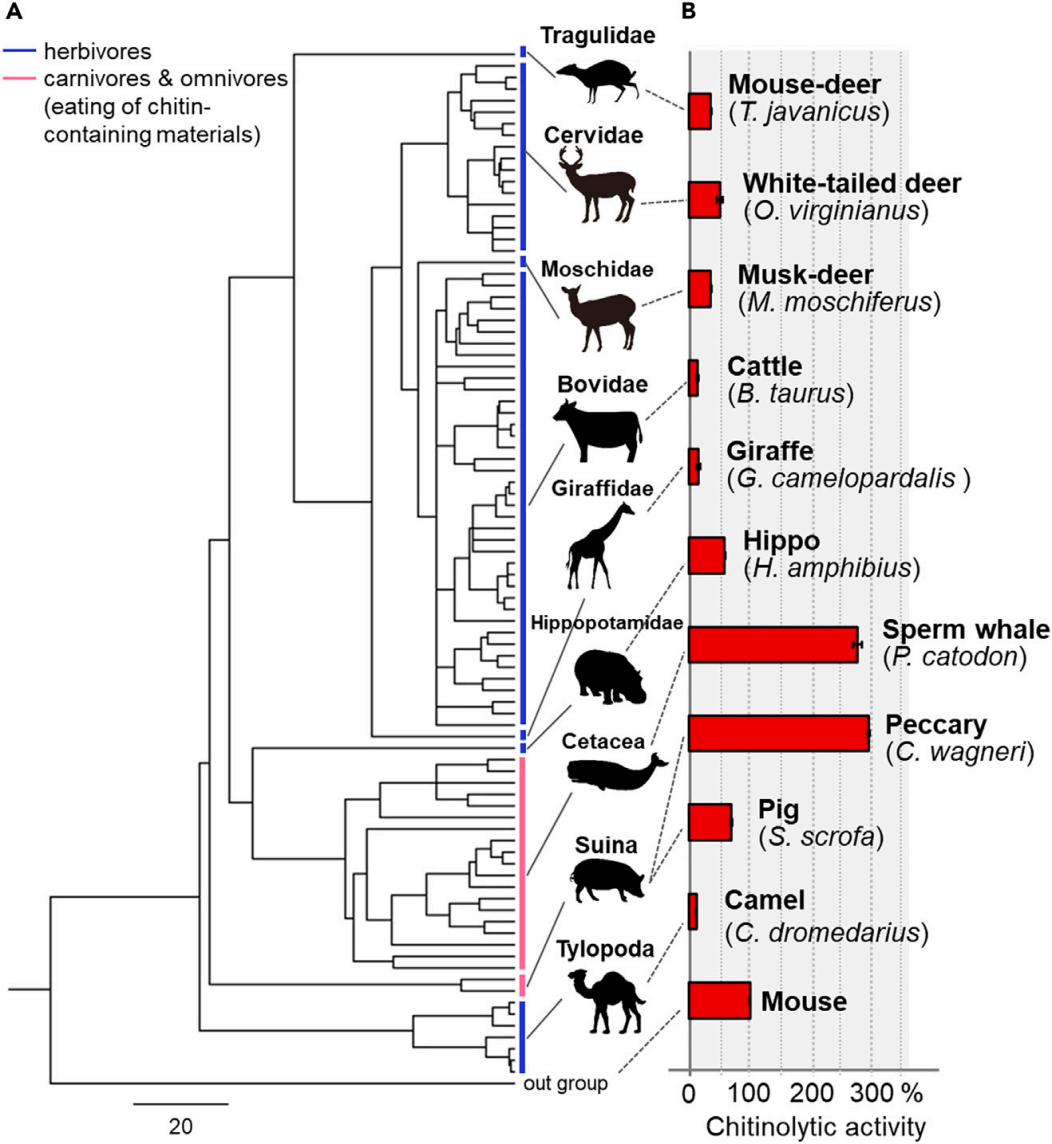
Inactivating events and enzyme activities of herbivorous Chia gene across the Cetartiodactyla phylogeny (A) Phylogenetic tree of 89 species from Cetartiodactyla with the mouse (outgroup). Phylogenetic relationships and divergence times were obtained from TimeTree v3.0 (http://www.timetree.org/). (B) The comparison of the chitinolytic activities of Chia proteins. The relative activity when the mouse Chia activity level at pH 2.0 was set to 100% is shown. Error bars represent mean ± SD from a single experiment conducted in triplicate. The illustrations (copyright AC Works Co., Ltd.) are used with permission.

To further investigate the molecular evolution of the *Chia* gene in herbivores, additional 47 species covering all major families of Cetartiodactyla were added to the analysis (Figure 4A; Tables S1 and S2). Within the CODEML program in the Phylogenetic Analysis by Maximum Likelihood (PAML) package,^45^ we used two pairs of branch models to test whether *Chia* in herbivorous branches is subject to positive selection. We evaluated the fit of the following branch models to the data: (1) the null one-ratio model (M0), which assumes the same ω value for all branches; and (2) a two-ratio model (M2), in which two different ω values were estimated for herbivorous (ω_foreground_) and omnivorous/carnivorous (ω_background_) branches were allowed to have different ω. We then compared the fit of models using likelihood ratio tests (LRTs). Unexpectedly, herbivores had lower ω (ω_foreground_ = 0.360) than the background ω of species with a non-herbivorous diet (ω_background_ = 0.430). However, M2 did not show better fitness than M0 (*χ*^*2*^ = 2.8, p = 0.424), indicating no significant difference in the strength of functional constraints between herbivorous and omnivorous/carnivorous species (Table 1).

**Table 1:** Estimated parameters of relaxed selection tests using branch models in CODEML

Model	ω_background_	ω_foreground_	*κ*	TL	np	log L	LR	*p*
M0 (null): one ratio model	0.385	0.385	4.01	3.57	174	−11248		
M2: two-branch classes	0.430	0.360	4.00	3.57	177	−11247	2.8	0.424

ω, a ratio of nonsynonymous to synonymous substitutions; *κ*, transition/transversion rate; TL, tree length; np, number of parameters; log L, log likelihood; LR, likelihood ratio; p, p value of the likelihood ratio test of M0 vs. M2 model.

We also used the RELAX program to test whether the strength of selection purification at these sites differed between the phylogenetic branches.^46^ RELAX results supported the hypothesis that selection was relaxed in *Chia* with herbivorous species (*k* = 0.45, p < 0.001, LR = 130.33, Table 2).

**Table 2:** Estimated parameters of relaxed selection using RELAX

Test branches	Reference branches	Model	log L	np	*k*	AIC_c_	LR	*p*
Herbivores	Omnivores	Null	−11263	63	1	22653	-	-
		Alternative	−11257	64	0.00	22642	13.05	0.000

log L, log-likelihood; np, number of parameters; AICc, sample size-corrected Akaike Information Criterion; *k*, selection intensity; LR, likelihood ratio; p, p value of likelihood ratio of alternative relative to null for each test.

### Chitinase activity of *Chia* in Cetartiodactyla lineages

To determine whether the functional changes in Chia are associated with different evolutionary characteristics, we determined chitinase activity in ten Cetartiodactyla species: mouse-deer (*Tragulus javanicus*), white-tailed deer (*Odocoileus virginianus*), musk-deer (*Moschus moschiferus*), cattle (*Bos taurus*), giraffe (*Giraffa camelopardalis*), hippo (*Hippopotamus amphibius*), sperm whale (*Physeter catodon*), peccary (*Catagonus wagneri*), pig (*Sus scrofa*), and Arabian camel (*Camelus dromedarius*) (Figures 4B and S10–S12). The pig, peccary, and sperm whale Chia showed 75–300% activity of the mouse enzyme and was significantly higher than in herbivorous species. In contrast, the mouse-deer, deer, musk-deer, and hippo Chia showed 40% activity, and Arabian camel and giraffe Chia showed <20% of the mouse enzyme (Figures 4B and S10–S12). These results suggested that the chitin-degrading activity of Chia was strongly associated with the feeding behavior of the Cetartiodactyla group.

### Chia5 has no complementary paralogs in ruminants

**Figure 5:**
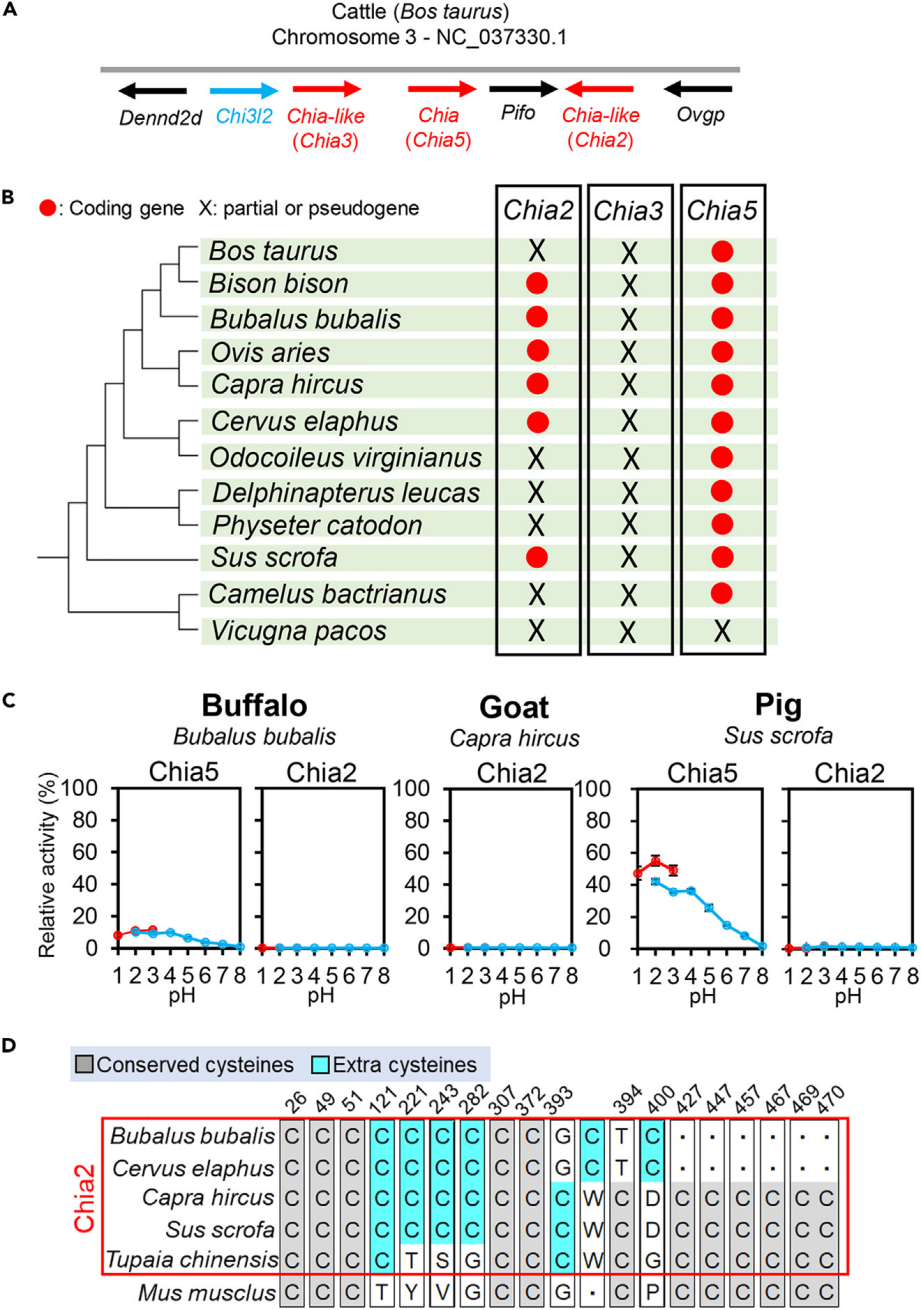
Activity of Chia paralogs in Cetartiodactyla (A) Schematic representation of the *Chia* paralogs (red) and neighboring marker genes, *Dennd2d*, *Pifo*, and *Ovgp* (black) in the cattle genome. Three Chia paralogs, named Chia-like, Chia, and Chia-like in the NCBI gene database, correspond to Chia3, Chia2, and Chia5, respectively.^33^ (B) Conservation of *Chia* paralogs in representative Cetartiodactyla. (C) The chitinase activity of buffalo Chia5 and Chia2, goat Chia2, and pig Chia2. The relative activity when the mouse Chia5 activity level at pH 2.0 was set to 100% is shown. Error bars represent mean ± SD from a single experiment conducted in triplicate. (D) The partial alignments of the Chia2 of Cetartiodactyla with tree shrew Chia2 and mouse Chia5.

Emerling et al. reported that modern mammals possess up to five *Chia* paralogs (*Chia1*-*Chia5*).^33^ The Chia molecule analyzed in this study (Figures 1, 2, 3, and 4) and mouse Chia correspond to the Chia5 paralog. Furthermore, the NCBI Gene database indicates that the cattle genome contains two *Chia* genes corresponding to *Chia2* (Gene ID: 786961) and *Chia3* (Gene ID: 101903127) (Figure 5A). However, *Chia2* is considered a pseudogene in cattle, and similar to *Chia3* that has three stop codons in the ORF. Investigation of the conservation of *Chia2* in the Cetartiodactyla genomes revealed that some species of Bovidae, Cervidae, and Suina possess genes that encode for CatD or full-length chitinase consisting of the CatD and chitin-binding domain (CBD) (Figure 5B; Table S3).

Hence, we investigated whether the *Chia2* genes found in certain species, including buffalo, goat, and pig, could compensate for the limited chitinase activity of Chia5. To accomplish this, we expressed these Chia2 and buffalo Chia5 in *E. coli*, as previously described (Figures 5C, S13, and S14). We found that buffalo Chia5 (histidine at position 128), has weak activity, similar to cattle, goat, and sheep Chia5 (Figure 5C). On the other hand, and in correspondence to previous reports, omnivorous pig Chia5 showed high activity (Figure 5C). Furthermore, Chia2 exhibited only faint activity in all analyzed species (Figure 5C).

We performed a comparative analysis of the amino acid sequences of mouse Chia (Chia5) with Chia2 of Cetartiodactyla and *Tupaia chinensis* (tree shrew). This species feeds on insects and has five *Chia* paralogs. The results revealed that the catalytic motif (DxxDxDxE) is conserved in all Chia2 proteins. However, we discovered additional cysteine residues in Chia2: two in tree shrew, five in pig and goat, and six in buffalo and deer (Figure 5D). These data suggest that Chia2 is inactivated in Cetartiodactyla regardless of the diet.

## Discussion

This study has identified a specific amino acid, H128, responsible for the evolutionary activity reduction of cattle Chia (Figures 1 and 2). This histidine residue is conserved in most bovids, whereas the insect-eating species have an arginine residue at position 128 (Figures 3 and 4). This observation suggested that substituting histidine with arginine could alleviate the functional constraints of Chia. However, despite several *Chia* paralogs in mammals, there is no compensation for the low active Chia5 (Figure 5).

The ruminants are one of the most successful mammalian lineages, exhibiting extensive morphological and ecological diversity. Recently, large-scale genome analysis of ruminants has been clarified, and genetic characteristics related to the diet and metabolism of ruminants have been reported.^47^ However, more progress has yet to be made in analyzing how these changes affect the function of each molecule. Here, we showed that functional constraints to *Chia* had been relaxed in the branches of ruminants in Cetartiodactyla. In these lineages, we also showed that only herbivorous Chia had a marked decrease in chitinase activity. Our results provided novel insights into ruminants’ evolution associated with feeding behavior.

Here we analyzed the enzymatic activity of Chia from eight ruminant species and showed that all the enzymes tested were reduced, but there were differences in activity levels. In Bovidae, the replacement of H128R significantly increased the activity of cattle Chia. Nevertheless, the sheep and goat Chia were not as active as that of cattle (Figure 3C). In addition, ruminant Chia other than Bovidae and giraffe showed lower activity than a mouse, even though they retained R128 (Figure 4B). In contrast, carnivorous whale and omnivorous peccary showed markedly higher activity than other ruminants, which was about 300% of that of the mouse (Figure 4B). Since these animals include chitin-containing feeds such as insects and crustaceans in their diet, they are thought to possess high chitinase activity. These results indicate that changes in the functional sites of Chia in Cetartiodactyla have evolved in a diverse and complex manner in each clade.

**Figure 6:**
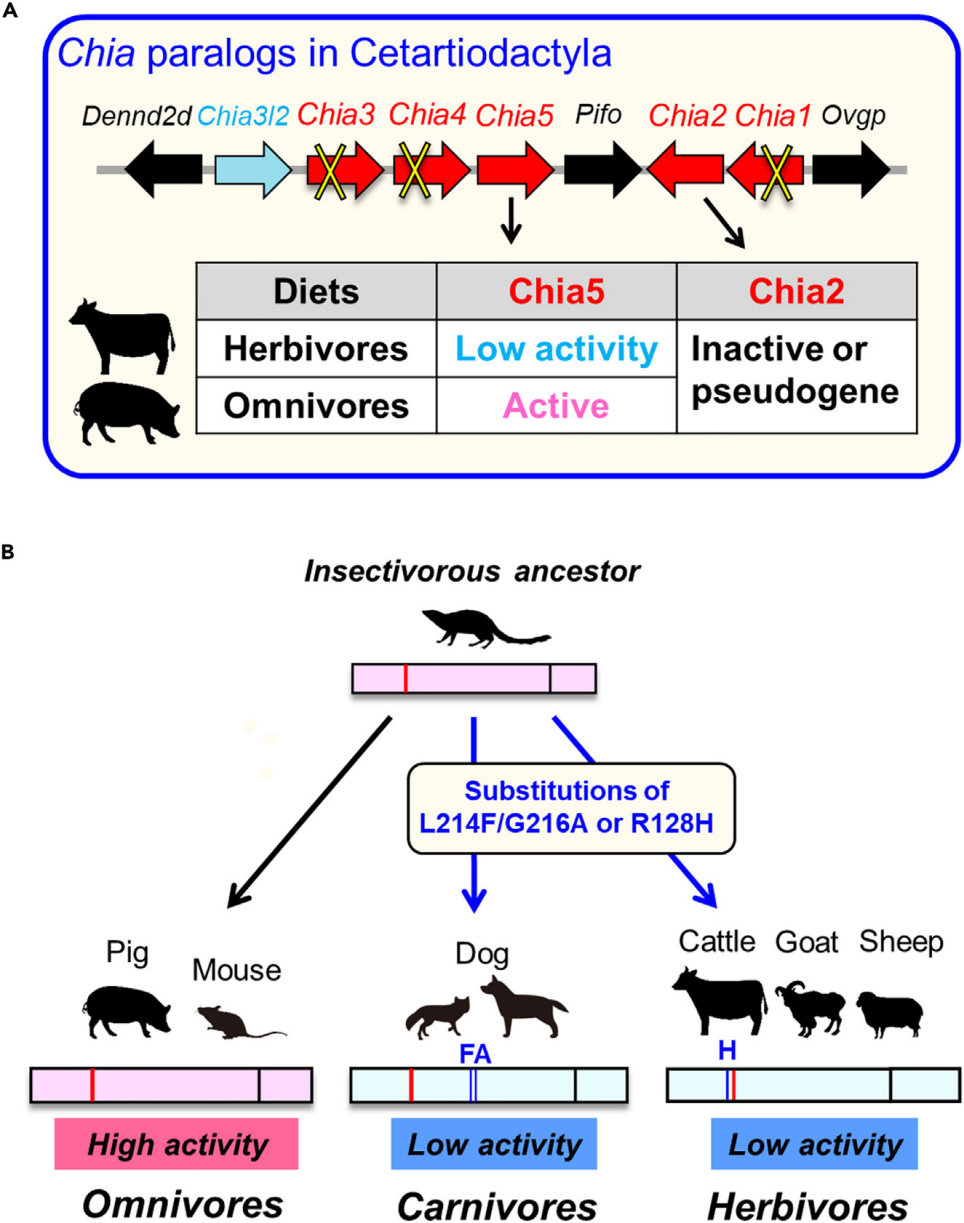
Evolution of Chia in modern mammals (A) Summary of the evolution of Chia paralogs in Cetartiodactyla. Chia4, Chia1, and Chia3 were pseudogenized outside or within this lineage.^33^ Chia2 is conserved in some species but was inactivated by the accumulation of cysteine mutations. Chia5, preserved as an intact gene, can be divided into active (omnivores) or low activity (herbivores) molecules according to their diets. (B) Summary of the Chia evolution in modern mammals that evolved from an insectivorous ancestor. Pig and mouse (omnivores) retain highly active Chia (left). Dog (carnivores) and cattle, goat, and sheep (herbivores) were inactivated by amino acid mutations at positions 214/216 or 128 due to the relaxation of functional constraints on Chia. The illustrations in (A) and (B) (copyright AC Works Co., Ltd.) are used with permission.

Emerling et al. reported the presence of up to five *Chia* paralogs in mammalian genomes, and that *Chia5* is the only functional paralog in some species such as cattle, mouse, and dog.^33^ Although almost all other *Chia* paralogs have also been lost in most Cetartiodactyla, *Chia2* is still functional at least in *Bubalus bubalis*, *Capra hircus*, and *Sus scrofa*. This gene may also be involved in chitin digestion in the species where it could potentially compensate for the generally reduced chitinolytic activity. However, although *Chia2* genes are relatively intact, the proteins contain extra cysteines and show little activity (Figures 5C and 5D). These observations are consistent with our previous reports on the inhibitory effect of extra cysteine residues.^35^ Thus, Chia2 is a pseudogenized and largely non-functional molecule in this phylogenetic group, regardless of the diet (Figure 6A).

The ancestors of all placental mammals were small insect-eating organisms that evolved shortly after the extinction of dinosaurs.^1^ It has been reported that *Chia* is a molecular record of the evolutionary process of the ancestors of insect-eating mammals.^33^ Chia enzymes from omnivorous animals such as mouse, pig, marmoset, and crab-eating monkey show high chitinolytic activity (Figure 6B, left).^23^^,^^24^^,^^25^^,^^30^^,^^31^ Our recent analysis of 32 carnivoran *Chia* genes showed that in non-insect-eating species, Chia had been inactivated or pseudogenized. In contrast, insect-eating species preserved the complete ORF and high activity of the enzyme (Figure 6B, middle).^35^ As described previously, the insect-eating bovids have arginine, while most bovids have histidine at position 128 (Figure 2; Figure 6B, right). The R128H substitution is also observed in some carnivorous *Chia* pseudogenes, including bears, mustelids, and pinnipeds.^35^ Previous studies have shown that Chia in carnivorous and herbivorous animals, having chitin-free diets, underwent genetic alterations and evolved into a low-active molecule while, the chitin-consuming species have maintained Chia with high activity (Figure 6B).

Chia expression and/or activity levels are markedly altered in various diseases, such as asthma and allergic inflammation.^10^^,^^11^^,^^18^^,^^19^ Chia-deficient mice accumulate chitin and develop age-dependent lung fibrosis, which can be ameliorated by Chia supplementation. This observation suggests that enhancing chitinase activity in Chia has therapeutic potential for reducing environmentally derived chitin in the lungs.^3^^,^^21^ We have reported that Chia activity in humans and dogs can be increased by R61M^19^ and F214L with A216G, respectively.^35^ In this study, we activated cattle Chia by replacing a single amino acid residue. Generally, our strategy for enzyme activation combines biochemical and evolutionary approaches. Such a strategy could, e.g., create a highly active Chia that can treat lung diseases.^21^^,^^48^

### Limitations of the study

While our study indicates the evolutionary activation of Chia in herbivores through the H128R mutation, it has certain limitations. This study focused only on the H128R mutation and its effect on the ruminant livestock Chia proteins. Further investigation on other potential mutations and their functional consequences in Chia across different herbivorous species is necessary to link the evolutionary structural and functional changes based on the specific diets. Extending such research would allow a comprehensive understanding of the evolutionary dynamics of Chia genes in herbivorous species.

## STAR★Methods

### Key resources table

**Table:** 

REAGENT or RESOURCE	SOURCE	IDENTIFIER
**Antibodies**		

Anti-V5-HRP monoclonal antibody	Thermo Fisher Scientific	R96125; RRID: AB_2556565

**Bacterial and virus strains**		

*E. coli* BL21(DE3) Competent Cells	Novagen (Merk)	69450

**Chemicals, peptides, and recombinant proteins**		

Bovine Total RNA Panel	Zyagen	BR-010
Pig Total RNA Panel	Zyagen	PR-010
Mouse Total RNA Master Panel	Takara Bio	636644
EcoRI	Takara Bio	1040A
XhoI	Takara Bio	1094A
Isopropyl β-D-thiogalactopyranoside (IPTG)	FUJIFILM Wako Pure Chemical Corporation	096-05143 · 4987481429178
Protease inhibitor (Complete)	Roche	04693132001
IgG Sepharose	Cytiva	17096901
4-methyl umbelliferyl β-D-*N, N′*-diacetyl chitobioside	Sigma-Aldrich	M9763-25MG
PD MidiTrap G-25	Cytiva	28918008

**Oligonucleotides**		

See Table S4 for a list of oligonucleotides		

**Recombinant DNA**		

Plasmid: pET22b/Protein A-Chia-V5-His	Tabata et al.^35^	N/A
Plasmid: pET22b/Protein A-mouse Chia-V5-His	This paper	N/A
Plasmid: pET22b/Protein A-cattle Chia-V5-His	This paper	N/A

**Software and algorithms**		

Nucleotide BLAST	NCBI	https://blast.ncbi.nlm.nih.gov/Blast.cgi
Genome	NCBI	https://www.ncbi.nlm.nih.gov/genome
MEGA X	Kumar et al.^50^	https://www.megasoftware.net/dload_win_gui
MUSCLE algorithm	Edgar et al.^51^	http://www.drive5.com/muscle
PAML	Yang^45^	http://abacus.gene.ucl.ac.uk/software/paml.html
RELAX	Wertheim et al.^46^	https://www.datamonkey.org/RELAX
TimeTree	Kumar et al.^52^	http://www.timetree.org/

**Other**		

GloMax Discover Multimode Microplate Reader	Promega	https://www.promega.com/products/microplate-readers-fluorometers-luminometers/microplate-readers/glomax-explorer-system/?catNum=GM3510&cs=y
Amersham ImageQuant 800 Western	Cytiva	https://www.cytivalifesciences.com/en/us/shop/protein-analysis/molecular-imaging-for-proteins/imaging-systems/amersham-imagequant-800-systems-p-11546

### Resource availability

#### Lead contact

Further information and requests for resources and reagents should be directed to and will be fulfilled by the lead contact, Fumitaka Oyama (f-oyama@cc.kogakuin.ac.jp).

#### Material availability

Materials generated in this study are available upon request. For further details contact the lead contact.

### Experimental model and subject details

We expressed proteins in *E. coli* as our experimental model instead of using living animals. We purchased total RNA products of bovine and porcine tissues from Zyagen (San Diego, CA, USA) to obtain the Chia cDNAs. These total RNA products are derived from normal healthy tissues for human consumption. The tissues were obtained from certified slaughterhouses in the USA and harvested from female donors at 30 months for bovine and 6 months for porcine. The animals used were domestic bovine and porcine animals specifically bred for meat production. We also purchased mouse stomach total RNA prepared from pooled healthy male/female BALB/c mice, ages 6 weeks, from Takara Bio (Mountain View, CA, USA). The use of animal-derived total RNAs and all procedures in this study were reviewed and approved by the Recombinant DNA Committee at Kogakuin University.

### Method details

#### Total RNA and cDNA preparation

The Cattle and Pig stomach Total RNA Panel were purchased from Zyagen. Mouse Total RNA Master Panel was also purchased from Takara Bio. Cattle, mouse, and pig stomach total RNAs were reverse-transcribed into cDNA essentially as described previously.^32^

#### Construction of Chia expression vector

We expressed mouse and cattle Chia as recombinant fusion proteins with pre-Protein A (PA) and V5-His (pEZZ18/PA-Chia-V5-His).^32^ In this report, PA-cattle Chia-V5-His and their derivative chimeric or mutant proteins were expressed by pET22b using the T7 promoter system (designated pET22b/Protein A-Chia-V5-His) as described recently.^35^

The pEZZ18/PA-cattle Chia-V5-His was digested with EcoRI and XhoI, generating cattle Chia cDNA. Fragments were purified and subcloned into similarly digested pET22b/pre-Protein A-mouse Chia-V5-His to produce pET22b/pre-Protein A-cattle Chia-V5-His.

#### Animal Chia cDNAs

Chia cDNAs encoding bush duiker, Harvey’s duiker, goat, sheep, mouse-deer, giraffe, hippo, sperm-whale, peccary, camel or buffalo were synthesized by Eurofins Genomics (Tokyo, Japan) with 5′-EcoRI and 3′-XhoI linker to produce pET22b/pre-Protein A-animal Chia protein-V5-His as described above.

#### Construction of expression plasmids for chimeric and mutant proteins and preparation of recombinant proteins

Mouse and cattle Chia have similar exon structures at the nucleotide level. To create mouse/cattle chimeric proteins, we fused two units at the junctions among exons 3–5, exons 6–7, exons 8–10 and exon 11 using template DNAs and primers (Figure S1; Tables S4 and S5) as described previously.^35^ More chimeras were also produced by combining templates and primers (Tables S4 and S5). Chia mutant proteins were prepared by PCR using a template and primers (Tables S4 and S5).^35^

*E. coli* BL21 (DE3) was transformed to express pre-Protein A-Chia-V5-His proteins using the plasmid DNAs. Transformed *E. coli* were grown in 250 mL of LB medium containing 100 μg/mL ampicillin at 37°C for 18 h. After induction with 0.1 mM isopropyl β-D-thiogalactopyranoside (IPTG), the bacteria were cultured for two h in an LB medium. Cells were harvested by centrifugation at 6,500 g for 20 min at 4°C. The recombinant protein was prepared from *E. coli* and purified by IgG Sepharose (Cytiva, Marlborough, MA, USA) chromatography as described previously.^35^ The protein-containing fractions were desalted using PD MidiTrap G-25 (Cytiva) equilibrated with TS buffer [20 mM Tris-HCl (pH 7.6), 150 mM NaCl and a protease inhibitor (Complete, Roche, Basel, Switzerland)]. Western blot detected recombinant products using an anti-V5-HRP monoclonal antibody (Thermo Fisher Scientific, Waltham, MA, USA).

#### Chitinase assays

We determined the chitinolytic activity using a synthetic fluorogenic substrate, 4-methyl umbelliferyl β-D-*N*, *N*′-diacetyl chitobioside [4-MU-(GlcNAc)_2_] (Sigma-Aldrich), as described previously.^35^ We measured the fluorescence of liberated 4-methyl umbelliferon using a GloMax Discover Multimode Microplate Reader (Promega, Madison, WI, USA) with excitation at 365 nm and emission at 445 nm.

To determine the optimal pH for chitinase activity, the enzyme was incubated with the 4-MU-(GlcNAc)_2_ substrate in 0.1 M Gly-HCl buffer (pH 1.0–3.0) or McIlvaine’s buffer (0.1 M citric acid and 0.2 M Na_2_HPO_4_; pH 2.0–8.0) at 37°C for 30 min.

### Quantification and statistical analysis

We quantified the immune blots using the Luminescent Image Analyzer (Amersham ImageQuant 800 Western, Cytiva, Marlborough, MA, USA) according to the manufacturer’s instructions. Welch’s t-test was used to compare the biochemical data. We carried out experiments in triplicate for statistical analysis.

#### Sequence analysis

We conducted NCBI BLAST searches against whole genome assemblies of 33 Bovidae, 1 Moschidae, 14 Cervidae, 1 Giraffidae, 1 Tragulidae, 4 Tylopoda, 1 Tayassuidae, 1 Hippopotamidae, and 15 Cetacea genomes from the NCBI Genome Database using the cattle (NM_174699.2), deer (XM_043878569.1), camel (XM_031457795.1), pig (NM_001258377.1), and whale (XM_030869341.1) *Chia* gene sequences as a query. In addition to these sequences, we used annotated gene sequences available in GenBank. GenBank accession numbers and deduced *Chia* nucleotide sequences are described in Tables S1 and S2 and Data S1. We imported all sequences, including the mouse as an outgroup, into MEGA X^49^ and aligned them using the MUSCLE algorithm.^50^ The evolutionary relationships of the *Chia* genes in Cetartiodactyla were estimated by the maximum-likelihood method (Figure S15).

#### Molecular evolution analysis

We performed positive selection analyses of genes based on a variation in the ratios of nonsynonymous to synonymous nucleotide substitutions (dN/dS or ω) using the CODEML program of PAML^45^ and RELAX implemented in HyPhy version 2.22 ^46^. The 88 Cetartiodactyla and mouse (outgroup) phylogenetic relationships were inferred using TimeTree (http://www.timetree.org/).^51^

For the CODEML analysis, we used branch models, where ω is assumed to be different between foreground and background branches. We first set up a null model estimating a single ω across all branches (M0). We then classified the tree into two distinct classes (M2): those with herbivores (foreground branches) and those with non-herbivores (background branches). Its goodness-of-fit was analyzed using likelihood ratio tests (LRTs).

We also used the RELAX program to test for two different rates of ω between lineages with herbivores versus all other branches and to distinguish positive from the relaxed selection because increased ω may indicate either.^46^ RELAX estimates ω among three rate classes for each branch using a branch site-random effects likelihood (BS-REL) model and then fits a parameter *k* indicating the strength of selection. Intensified selection is indicated by *k* > 1, whereas relaxed selection is indicated by *k* < 1. The goodness-of-fit for two given models was analyzed using the LRT by comparison with each null model whose *k* parameter was constrained to 1.

## Data Availability

• All data reported in this paper will be shared by the lead contact upon request. • This paper does not report original code. • Any additional information required to reanalyze the data reported in this paper is available from the lead contact upon request.

## Supplemental information

Document S1. Figures S1–S15

Table S1. NCBI Gene Accession numbers of Chia, related to Figures 3 and 4

Table S2. NCBI Genome Accession numbers for Chia sequences, related to Figures 3 and 4

Table S3. NCBI Gene ID and proteins of Chia paralogs, related to Figure 5

Table S4. Construction of chimeric and mutant proteins by PCR, related to STAR Methods

Table S5. Primers for construction of chimeric and mutant proteins, related to STAR Methods

Data S1. Nucleotide sequences of 41 bovid Chia to construct the phylogenetic tree, related to Figure 3

## Footnotes

Supplemental information can be found online at https://doi.org/10.1016/j.isci.2023.107254.
