# Supplementary material for: Evolutionary activation of acidic chitinase in herbivores through the H128R mutation in ruminant livestock
Source: iScience. 2023 Jul 3;26(8):107254. doi: 10.1016/j.isci.2023.107254 (PMC10368815; doi:10.1016/j.isci.2023.107254)
Supplement: Document S1. Figures S1–S15 [file mmc1.pdf]

iScience, Volume *26*

Supplemental information

Evolutionary activation of acidic chitinase in herbivores through the H128R mutation in ruminant livestock

Eri Tabata, Ikuto Kobayashi, Takuya Morikawa, Akinori Kashimura, Peter O. Bauer, and Fumitaka Oyama

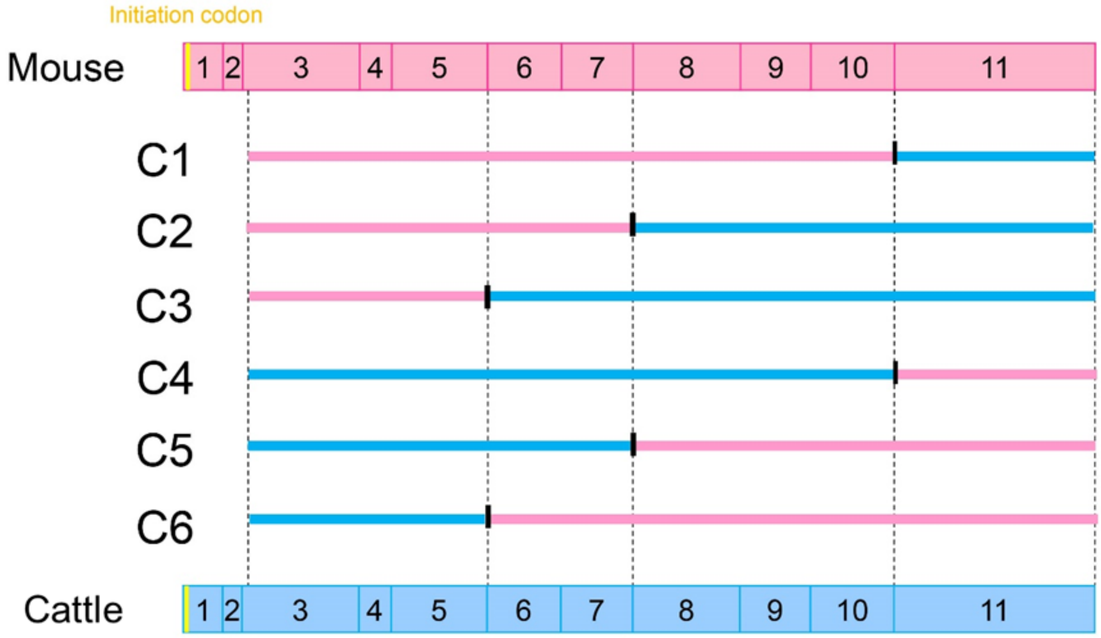

Figure S1. Prepared mouse-cattle chimeric Chia proteins, related to Figure 1. The numbers represent the exons.

Mouse Chia

```
YNLICYFTNWAQYRPGLGSFKPDDINPCLCTHLIYAFAGMQNNEITTIEWNDVTLYKAFNDLKN
RNSKLKTLLAIGGWNFGTAPFTTMVSTSQNRQTFITSVIKFLRQYGFDGLDLDWEYPGSRGSPP
QDKHLFTVLVKEMREAFEQEAIESNRPRLMVTAAVAGGISNIQAGYEIPELSKYLDFIHVMTYD
LHGSWEGYTGENSPLYKYPTETGSNAYLNVDYVMNYWKNNGAPAEKLIVGFPEYGHTFILRNPS
DNGIGAPTSGDGPAGPYTRQAGFWAYYEICTFLRSGATEVWDASQEVPYAYKANEWLGYDNIKS
FSVKAQWLKQNNFGGAMIWAIDLDDFTGSFCDQGKFPLTSTLNKALGISTEGCTAPDVPSEPVT
TPPGSGSGGGSSGGSSGGSGFCADKADGLYPVADDRNAFWQCINGITYQQHCQAGLVFDTSCNC
CNWP
```

Cattle Chia

```
YQLVCYFSNWAQYRPGLGSFKPDNIDPCLCTHLIYAFAGMSNSEITTIEWNDVALYSSFNDLKK
KNSQLKILLAIGGWNFGTAPFTAMVATPENRKTFISSVIKFLHQYGFDGLDFDWEYPGFRGSPS
QDKHLFTVLVQETREAFEQEAKQTNKPRLLVTAAVAAGISNIQAGYEIPQLSQYLDFIHVMTYD
FHGSWEGYTGENSPLYKYPTDTGSNTYLNVEYAMNYWKKNGAPAEKLIIGFPAYGHNFILRDAS
NNGIGAPTSGAGPAGPYTREAGFWAYYEICAFLKDGATEAWDDSQNVPYAYKGTEWVGYDNVNS
FRIKAQWLKENNFGGAMVWAIDLDDFTGTFCNQGKFPLINTLKDALGLKSATCNASTQSSEPNS
SPGNESGSGNKSSSSEGRGYCAGKADGLYPVADNRNAFWNCVNGITYKQNCLTGLVFDTSCHCC
NWA
```

Figure S2. Deduced amino acid sequences of the recombinant mouse and cattle Chia proteins expressed in *E. coli*, related to Figure 1*.* The amino acid sequences are color-coded, consistent with Figure 1.

Chimera C1

YFTNWAQYRPGLGSFKPDDINPCLCTHLIYAFAGMQNNEITTIEWNDVTLYKAFNDLKNRNSKL
KTLLAIGGWNFGTAPFTTMVSTSQNRQTFITSVIKFLRQYGFDGLDLDWEYPGSRGSPPQDKHL
FTVLVKEMREAFEQEAIESNRPRLMVTAAVAGGISNIQAGYEIPELSKYLDFIHVMTYDLHGSW
EGYTGENSPLYKYPTETGSNAYLNVDYVMNYWKNNGAPAEKLIVGFPEYGHTFILRNPSDNGIG
APTSGDGPAGPYTRQAGFWAYYEICTFLRSGATEVWDASQEVPYAYKANEWLGYDNIKSFSVKA
QWLKQNNFGGAMIWAIDLDDFTGSFCDQGKFPLTSTLNKALGISTEGCNASTQSSEPNSSPGNE
SGSGNKSSSSEGRGYCAGKADGLYPVADNRNAFWNCVNGITYKQNCLTGLVFDTSCHCCNWA

Chimera C2

YNLICYFTNWAQYRPGLGSFKPDDINPCLCTHLIYAFAGMQNNEITTIEWNDVTLYKAFNDLKN
RNSKLKTLLAIGGWNFGTAPFTTMVSTSQNRQTFITSVIKFLRQYGFDGLDLDWEYPGSRGSPP
QDKHLFTVLVKEMREAFEQEAIESNRPRLMVTAAVAGGISNIQAGYEIPELSKYLDFIHVMTYD
LHGSWEGYTGENSPLYKYPTETGSNAYLNVEYAMNYWKKNGAPAEKLIIGFPAYGHNFILRDAS
NNGIGAPTSGAGPAGPYTREAGFWAYYEICAFLKDGATEAWDDSQNVPYAYKGTEWVGYDNVNS
FRIKAQWLKENNFGGAMVWAIDLDDFTGTFCNQGKFPLINTLKDALGLKSATCNASTQSSEPNS
SPGNESGSGNKSSSSEGRGYCAGKADGLYPVADNRNAFWNCVNGITYKQNCLTGLVFDTSCHCC
NWA

Chimera C3

YNLICYFTNWAQYRPGLGSFKPDDINPCLCTHLIYAFAGMQNNEITTIEWNDVTLYKAFNDLKN
RNSKLKTLLAIGGWNFGTAPFTTMVSTSQNRQTFITSVIKFLRQYGFDGLDLDWEYPGSRGSPP
QDKHLFTVLVKETREAFEQEAKQTNKPRLLVTAAVAAGISNIQAGYEIPQLSQYLDFIHVMTYD
FHGSWEGYTGENSPLYKYPTDTGSNTYLNVEYAMNYWKKNGAPAEKLIIGFPAYGHNFILRDAS
NNGIGAPTSGAGPAGPYTREAGFWAYYEICAFLKDGATEAWDDSQNVPYAYKGTEWVGYDNVNS
FRIKAQWLKENNFGGAMVWAIDLDDFTGTFCNQGKFPLINTLKDALGLKSATCNASTQSSEPNS
SPGNESGSGNKSSSSEGRGYCAGKADGLYPVADNRNAFWNCVNGITYKQNCLTGLVFDTSCHCC
NWA

Chimera C4

YQLVCYFSNWAQYRPGLGSFKPDNIDPCLCTHLIYAFAGMSNSEITTIEWNDVALYSSFNDLKK
KNSQLKILLAIGGWNFGTAPFTAMVATPENRKTFISSVIKFLHQYGFDGLDFDWEYPGFRGSPS
QDKHLFTVLVQETREAFEQEAKQTNKPRLLVTAAVAAGISNIQAGYEIPQLSQYLDFIHVMTYD
FHGSWEGYTGENSPLYKYPTDTGSNTYLNVEYAMNYWKKNGAPAEKLIIGFPAYGHNFILRDAS
NNGIGAPTSGAGPAGPYTREAGFWAYYEICAFLKDGATEAWDDSQNVPYAYKGTEWVGYDNVNS
FRIKAQWLKENNFGGAMVWAIDLDDFTGTFCNQGKFPLINTLKDALGLKSATCTAPDVPSEPVT
TPPGSGSGGGSSGGSSGGSGFCADKADGLYPVADDRNAFWQCINGITYQQHCQAGLVFDTSCNC
CNWP

Chimera C5

YQLVCYFSNWAQYRPGLGSFKPDNIDPCLCTHLIYAFAGMSNSEITTIEWNDVALYSSFNDLKK
KNSQLKILLAIGGWNFGTAPFTAMVATPENRKTFISSVIKFLHQYGFDGLDFDWEYPGFRGSPS
QDKHLFTVLVQETREAFEQEAKQTNKPRLLVTAAVAAGISNIQAGYEIPQLSQYLDFIHVMTYD
FHGSWEGYTGENSPLYKYPTDTGSNTYLNVDYVMNYWKNNGAPAEKLIVGFPEYGHTFILRNPS
DNGIGAPTSGDGPAGPYTRQAGFWAYYEICTFLRSGATEVWDASQEVPYAYKANEWLGYDNIKS
FSVKAQWLKQNNFGGAMIWAIDLDDFTGSFCDQGKFPLTSTLNKALGISTEGCTAPDVPSEPVT
TPPGSGSGGGSSGGSSGGSGFCADKADGLYPVADDRNAFWQCINGITYQQHCQAGLVFDTSCNC
CNWP

Chimera C6

YQLVCYFSNWAQYRPGLGSFKPDNIDPCLCTHLIYAFAGMSNSEITTIEWNDVALYSSFNDLKK
KNSQLKILLAIGGWNFGTAPFTAMVATPENRKTFISSVIKFLHQYGFDGLDFDWEYPGFRGSPS
QDKHLFTVLVQEMREAFEQEAIESNRPRLMVTAAVAGGISNIQAGYEIPELSKYLDFIHVMTYD
LHGSWEGYTGENSPLYKYPTETGSNAYLNVDYVMNYWKNNGAPAEKLIVGFPEYGHTFILRNPS
DNGIGAPTSGDGPAGPYTRQAGFWAYYEICTFLRSGATEVWDASQEVPYAYKANEWLGYDNIKS
FSVKAQWLKQNNFGGAMIWAIDLDDFTGSFCDQGKFPLTSTLNKALGISTEGCTAPDVPSEPVT
TPPGSGSGGGSSGGSSGGSGFCADKADGLYPVADDRNAFWQCINGITYQQHCQAGLVFDTSCNC
CNWP

Figure S3. Deduced amino acid sequences of the chimeric Chia proteins C1-C6 expressed in *E. coli*, related to Figure 1*.* The amino acid sequences are color-coded, consistent with Figure 1. In chimeras, pink, mouse Chia; blue, cattle Chia.

(A)

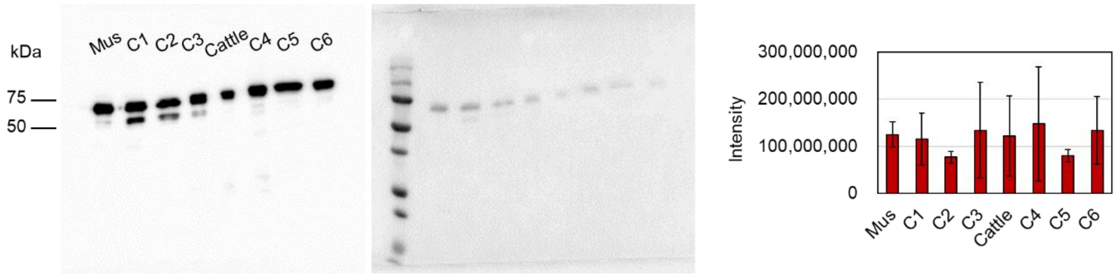

(B)

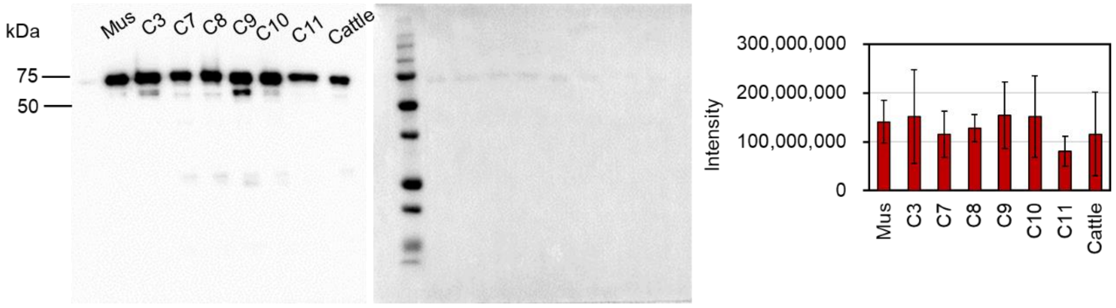

(C)

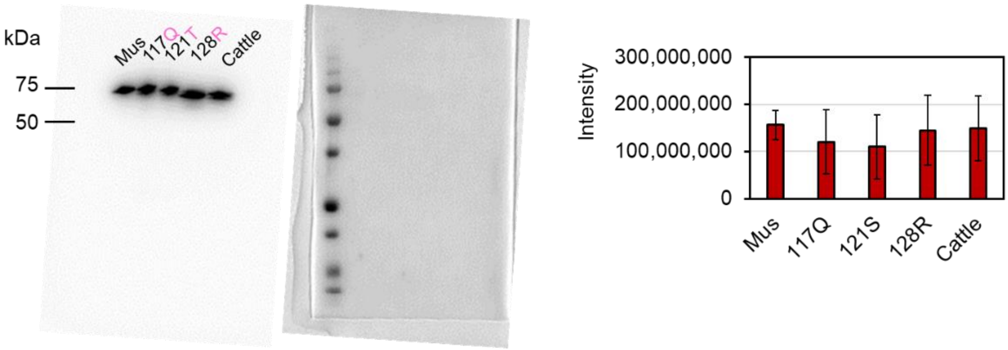

Figure S4. Western blot analysis of the recombinant proteins, related to Figures 1 and 2. Each gel image and graph corresponds to the figure in the main text as follows: (A), Figure 1B; (B), Figure 1D and Figure 2B; (C), Figure 2E. We analyzed the protein fractions using standard SDS-PAGE, followed by Western blot using an anti-V5 antibody. Full-length gel images of Western blots (left panels) and molecular weight markers (middle panels). The signal intensities were quantified in the Star Methods (right panels).

Chimera C7
YNLICYFTNWAQYRPGLGSFKPDDINPCLCTHLIYAFAGMQNNEITTIEWNDVTLYKAFNDLKN
RNSKLKTLLAIGGWNFGTAPFTAMVATPENRKTFISSVIKFLHQYGFDGLDFDWEYPGFRGSPS
QDKHLFTVLVQETREAFEQEAKQTNKPRLLVTAAVAAGISNIQAGYEIPQLSQYLDFIHVMTYD
FHGSWEGYTGENSPLYKYPTDTGSNTYLNVEYAMNYWKKNGAPAEKLIIGFPAYGHNFILRDAS
NNGIGAPTSGAGPAGPYTREAGFWAYYEICAFLKDGATEAWDDSQNVPYAYKGTEWVGYDNVNS
FRIKAQWLKENNFGGAMVWAIDLDDFTGTFCNQGKFPLINTLKDALGLKSATCNASTQSSEPNS
SPGNESGSGNKSSSSEGRGYCAGKADGLYPVADNRNAFWNCVNGITYKQNCLTGLVFDTSCHCC
NWA

Chimera C8
YNLICYFTNWAQYRPGLGSFKPDDINPCLCTHLIYAFAGMQNNEITTIEWNDVTLYKAFNDLKN
RNSQLKILLAIGGWNFGTAPFTAMVATPENRKTFISSVIKFLHQYGFDGLDFDWEYPGFRGSPS
QDKHLFTVLVQETREAFEQEAKQTNKPRLLVTAAVAAGISNIQAGYEIPQLSQYLDFIHVMTYD
FHGSWEGYTGENSPLYKYPTDTGSNTYLNVEYAMNYWKKNGAPAEKLIIGFPAYGHNFILRDAS
NNGIGAPTSGAGPAGPYTREAGFWAYYEICAFLKDGATEAWDDSQNVPYAYKGTEWVGYDNVNS
FRIKAQWLKENNFGGAMVWAIDLDDFTGTFCNQGKFPLINTLKDALGLKSATCNASTQSSEPNS
SPGNESGSGNKSSSSEGRGYCAGKADGLYPVADNRNAFWNCVNGITYKQNCLTGLVFDTSCHCC
NWA

Chimera C9
YQLVCYFSNWAQYRPGLGSFKPDNIDPCLCTHLIYAFAGMSNSEITTIEWNDVALYSSFNDLKK
KNSQLKILLAIGGWNFGTAPFTTMVSTSQNRQTFITSVIKFLRQYGFDGLDLDWEYPGSRGSPP
QDKHLFTVLVKETREAFEQEAKQTNKPRLLVTAAVAAGISNIQAGYEIPQLSQYLDFIHVMTYD
FHGSWEGYTGENSPLYKYPTDTGSNTYLNVEYAMNYWKKNGAPAEKLIIGFPAYGHNFILRDAS
NNGIGAPTSGAGPAGPYTREAGFWAYYEICAFLKDGATEAWDDSQNVPYAYKGTEWVGYDNVNS
FRIKAQWLKENNFGGAMVWAIDLDDFTGTFCNQGKFPLINTLKDALGLKSATCNASTQSSEPNS
SPGNESGSGNKSSSSEGRGYCAGKADGLYPVADNRNAFWNCVNGITYKQNCLTGLVFDTSCHCC
NWA

Chimera C10
YQLVCYFSNWAQYRPGLGSFKPDNIDPCLCTHLIYAFAGMSNSEITTIEWNDVALYSSFNDLKK
KNSQLKILLAIGGWNFGTAPFTTMVSTSQNRQTFITSVIKFLRQYGFDGLDFDWEYPGSRGSPS
HDKHLFTVLVQETREAFEQEAKQTNKPRLLVTAAVAAGISNIQAGYEIPQLSQYLDFIHVMTYD
FHGSWEGYTGENSPLYKYPTDTGSNTYLNVEYAMNYWKKNGAPAEKLIIGFPAYGHNFILRDAS
NNGIGAPTSGAGPAGPYTREAGFWAYYEICAFLKDGATEAWDDSQNVPYAYKGTEWVGYDNVNS
FRIKAQWLKENNFGGAMVWAIDLDDFTGTFCNQGKFPLINTLKDALGLKSATCNASTQSSEPNS
SPGNESGSGNKSSSSEGRGYCAGKADGLYPVADNRNAFWNCVNGITYKQNCLTGLVFDTSCHCC
NWA

Chimera C11
YQLVCYFSNWAQYRPGLGSFKPDNIDPCLCTHLIYAFAGMSNSEITTIEWNDVALYSSFNDLKK
KNSQLKILLAIGGWNFGTAPFTTMVSTSQNRKTFISSVIKFLHQYGFDGLDFDWEYPGFRGSPS
QDKHLFTVLVQETREAFEQEAKQTNKPRLLVTAAVAAGISNIQAGYEIPQLSQYLDFIHVMTYD
FHGSWEGYTGENSPLYKYPTDTGSNTYLNVEYAMNYWKKNGAPAEKLIIGFPAYGHNFILRDAS
NNGIGAPTSGAGPAGPYTREAGFWAYYEICAFLKDGATEAWDDSQNVPYAYKGTEWVGYDNVNS
FRIKAQWLKENNFGGAMVWAIDLDDFTGTFCNQGKFPLINTLKDALGLKSATCNASTQSSEPNS
SPGNESGSGNKSSSSEGRGYCAGKADGLYPVADNRNAFWNCVNGITYKQNCLTGLVFDTSCHCC
NWA

Figure S5. Deduced amino acid sequences of the recombinant Chia chimeric proteins C7-C11 expressed in *E. coli*, related to Figures 1 and 2. The amino acid sequences are color-coded, consistent with Figures 1 and 2. In chimeras, pink, mouse Chia; blue, cattle Chia.

Cattle 3Mut
YQLVCYFSNWAQYRPGLGSFKPDNIDPCLCTHLIYAFAGMSNSEITTIEWNDVALYSSFNDLKK
KNSQLKILLAIGGWNFGTAPFTAMVATPENRQTFITSVIKFLRQYGFDGLDFDWEYPGFRGSPS
QDKHLFTVLVQETREAFEQEAKQTNKPRLLVTAAVAAGISNIQAGYEIPQLSQYLDFIHVMTYD
FHGSWEGYTGENSPLYKYPTDTGSNTYLNVEYAMNYWKKNGAPAEKLIIGFPAYGHNFILRDAS
NNGIGAPTSGAGPAGPYTREAGFWAYYEICAFLKDGATEAWDDSQNVPYAYKGTEWVGYDNVNS
FRIKAQWLKENNFGGAMVWAIDLDDFTGTFCNQGKFPLINTLKDALGLKSATCNASTQSSEPNS
SPGNESGSGNKSSSSEGRGYCAGKADGLYPVADNRNAFWNCVNGITYKQNCLTGLVFDTSCHCC
NWA

Cattle_K117Q
YQLVCYFSNWAQYRPGLGSFKPDNIDPCLCTHLIYAFAGMSNSEITTIEWNDVALYSSFNDLKK
KNSQLKILLAIGGWNFGTAPFTAMVATPENRQTFISSVIKFLHQYGFDGLDFDWEYPGFRGSPS
QDKHLFTVLVQETREAFEQEAKQTNKPRLLVTAAVAAGISNIQAGYEIPQLSQYLDFIHVMTYD
FHGSWEGYTGENSPLYKYPTDTGSNTYLNVEYAMNYWKKNGAPAEKLIIGFPAYGHNFILRDAS
NNGIGAPTSGAGPAGPYTREAGFWAYYEICAFLKDGATEAWDDSQNVPYAYKGTEWVGYDNVNS
FRIKAQWLKENNFGGAMVWAIDLDDFTGTFCNQGKFPLINTLKDALGLKSATCNASTQSSEPNS
SPGNESGSGNKSSSSEGRGYCAGKADGLYPVADNRNAFWNCVNGITYKQNCLTGLVFDTSCHCC
NWA

Cattle_S121T
MYQLVCYFSNWAQYRPGLGSFKPDNIDPCLCTHLIYAFAGMSNSEITTIEWNDVALYSSFNDLK
KKNSQLKILLAIGGWNFGTAPFTAMVATPENRKTFITSVIKFLHQYGFDGLDFDWEYPGFRGSP
SQDKHLFTVLVQETREAFEQEAKQTNKPRLLVTAAVAAGISNIQAGYEIPQLSQYLDFIHVMTY
DFHGSWEGYTGENSPLYKYPTDTGSNTYLNVEYAMNYWKKNGAPAEKLIIGFPAYGHNFILRDA
SNNGIGAPTSGAGPAGPYTREAGFWAYYEICAFLKDGATEAWDDSQNVPYAYKGTEWVGYDNVN
SFRIKAQWLKENNFGGAMVWAIDLDDFTGTFCNQGKFPLINTLKDALGLKSATCNASTQSSEPN
SSPGNESGSGNKSSSSEGRGYCAGKADGLYPVADNRNAFWNCVNGITYKQNCLTGLVFDTSCHC
CNWA

Cattle_H128R
YQLVCYFSNWAQYRPGLGSFKPDNIDPCLCTHLIYAFAGMSNSEITTIEWNDVALYSSFNDLKK
KNSQLKILLAIGGWNFGTAPFTAMVATPENRKTFISSVIKFLRQYGFDGLDFDWEYPGFRGSPS
QDKHLFTVLVQETREAFEQEAKQTNKPRLLVTAAVAAGISNIQAGYEIPQLSQYLDFIHVMTYD
FHGSWEGYTGENSPLYKYPTDTGSNTYLNVEYAMNYWKKNGAPAEKLIIGFPAYGHNFILRDAS
NNGIGAPTSGAGPAGPYTREAGFWAYYEICAFLKDGATEAWDDSQNVPYAYKGTEWVGYDNVNS
FRIKAQWLKENNFGGAMVWAIDLDDFTGTFCNQGKFPLINTLKDALGLKSATCNASTQSSEPNS
SPGNESGSGNKSSSSEGRGYCAGKADGLYPVADNRNAFWNCVNGITYKQNCLTGLVFDTSCHCC
NWA

Figure S6. Deduced amino acid sequences of the mutant cattle Chia proteins expressed in *E. coli*, related to Figure 2*.* The amino acid sequences are color-coded, consistent with Figure 2. In mutants, pink, mouse Chia amino acids; blue, cattle Chia amino acids.

Harvey's duiker

YQLVCYFSNWAQYRPGLGSFKPDNIDPCLCTHLIYAFAGMSNSEITTTEWNDVALYSSFNDLKK
KNSQLKILLAIGGWNFGTAPFTAMVATPENRKTFISSVIKFLRQYGFDGLDFDWEYPGSNGSPS
QDKHLFTVLVQETREAFEQEAEQTNKPRLLVTAAVAAGISNIQAGYEIPQLSQYLDFIHVMTYD
LHGSWEGYTGENSPLYKYPTDTGSNAYLNVEYAINYWKNSGAPAEKLIVGFPAYGHNFILRDAS
NNGIGAPTSGAGPAGPYTREAGFWAYYEICTFLKDGATEAWDDSQKAPYAYKGTEWVGYDNVNS
FRIKAQWLKENNFGGAMVWAIDLDDFTGTFCNQGKFPLINTLKDALGLKSATCNTPTQTSEPNS
SPGNESGSGNESSSPESGGYCAGKADGLYPVADNRNAFWNCANGITYEQNCPTGLVFDTSCHCC
SWT

Bush duiker

YQLVCYFSNWAQYRPGLGSFKPDNIDPCLCTHLIYAFAGMSNSEITTTEWNDVALYSSFNDLKK
KNSQLKILLAIGGWNFGTAPFTAMVATPENRKTFISSVIKFLRQYGFDGLDFDWEYPGSNGSPS
QDKHLFTVLVQETREAFEQEAEQTNKPRLLVTAAVAAGISNIQAGYEIPQLSQYLDFIHVMTYD
LHGSWEGYTGENSPLYKYPTDTGSNAYLNVEYAINYWKNSGAPAEKLIVGFPAYGHNFILRDAS
NNGIGAPTSGAGPAGPYTREAGFWAYYEICTFLKDGATEAWDDSQKAPYAYKGTEWVGYDNVNS
FRIKAQWLKENNFGGAMVWAIDLDDFTGTFCNQGKFPLINTLKDALGLKSATCNTPTQTSEPNS
SPGNESGSGNESSSPESGGYCAGKADGLYPVADNRNAFWNCANGITYEQNCPTGLVFDTSCHCC
SWT

Harvey's duiker_128H

YQLVCYFSNWAQYRPGLGSFKPDNIDPCLCTHLIYAFAGMSNSEITTTEWNDVALYSSFNDLKK
KNSQLKILLAIGGWNFGTAPFTAMVATPENRKTFISSVIKFLHQYGFDGLDFDWEYPGSNGSPS
QDKHLFTVLVQETREAFEQEAEQTNKPRLLVTAAVAAGISNIQAGYEIPQLSQYLDFIHVMTYD
LHGSWEGYTGENSPLYKYPTDTGSNAYLNVEYAINYWKNSGAPAEKLIVGFPAYGHNFILRDAS
NNGIGAPTSGAGPAGPYTREAGFWAYYEICTFLKDGATEAWDDSQKAPYAYKGTEWVGYDNVNS
FRIKAQWLKENNFGGAMVWAIDLDDFTGTFCNQGKFPLINTLKDALGLKSATCNTPTQTSEPNS
SPGNESGSGNESSSPESGGYCAGKADGLYPVADNRNAFWNCANGITYEQNCPTGLVFDTSCHCC
SWT

Bush duiker_128H

YQLVCYFSNWAQYRPGLGSFKPDNIDPCLCTHLIYAFAGMSNSEITTTEWNDVALYSSFNDLKK
KNSQLKILLAIGGWNFGTAPFTAMVATPENRKTFISSVIKFLHQYGFDGLDFDWEYPGSNGSPS
QDKHLFTVLVQETREAFEQEAEQTNKPRLLVTAAVAAGISNIQAGYEIPQLSQYLDFIHVMTYD
LHGSWEGYTGENSPLYKYPTDTGSNAYLNVEYAINYWKNSGAPAEKLIVGFPAYGHNFILRDAS
NNGIGAPTSGAGPAGPYTREAGFWAYYEICTFLKDGATEAWDDSQKAPYAYKGTEWVGYDNVNS
FRIKAQWLKENNFGGAMVWAIDLDDFTGTFCNQGKFPLINTLKDALGLKSATCNTPTQTSEPNS
SPGNESGSGNESSSPESGGYCAGKADGLYPVADNRNAFWNCANGITYEQNCPTGLVFDTSCHCC
SWT

Figure S7. Deduced amino acid sequences of the wild and mutant duiker Chia proteins expressed in *E. coli*, related to Figure 3. The amino acid sequences are color-coded, consistent with Figure 3. Green, duiker Chia.

(A)

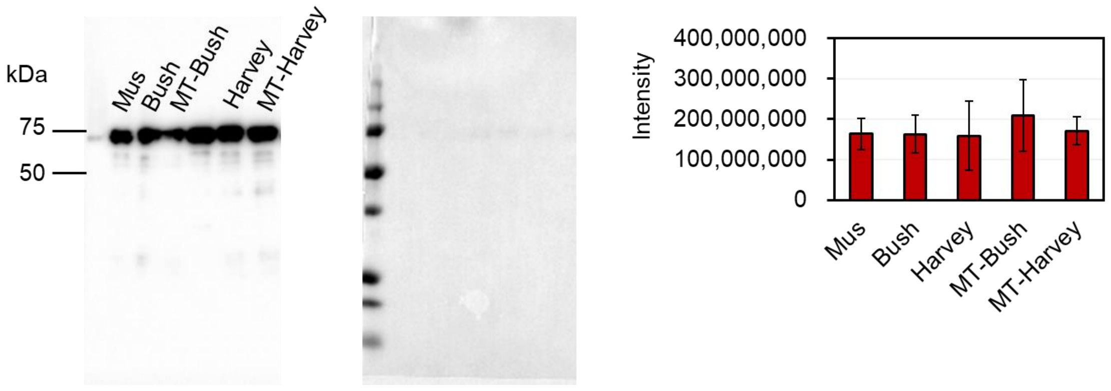

(B)

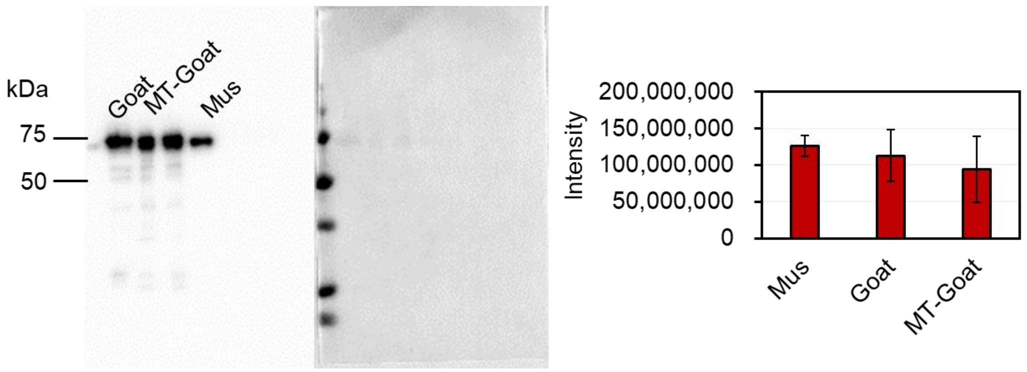

(C)

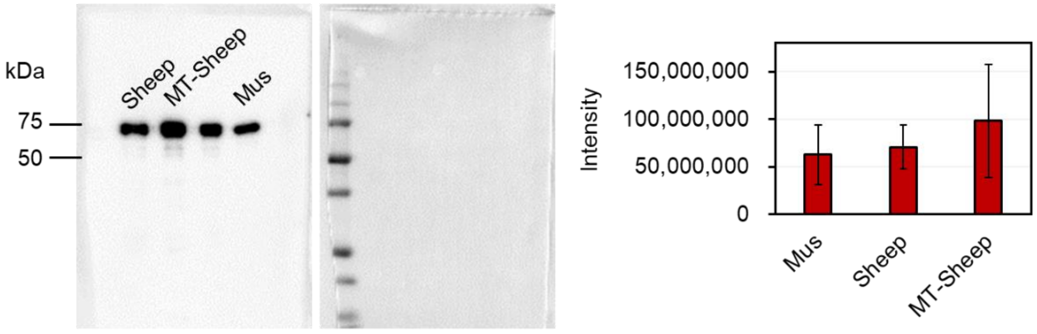

Figure S8. Western blot analysis of the recombinant proteins, related to Figure 3. Each gel image and graph corresponds to the figure in the main text as follows: (A), Figure 3B; (B), Figure 3C; (C), Figure 3C. We analyzed the protein fractions using standard SDS-PAGE, followed by Western blot using an anti-V5 antibody. Full-length gel images of Western blots (left panels) and molecular weight markers (middle panels). The signal intensities were quantified in the Star Methods (right panels).

Goat

```
YQLVCYFSNWAQYRPGLGSFKPDNIDPCLCTHLIYAFAGMSNSEITTIEWNDVALYSSFNDLKK
KNSQLKILLAIGGWNFGTAPFTAMVATPENRKTFISSVIKFLHQYGFDGLDFDWEYPGSRGSPS
QDKHLFTVLVQETREAFEQEAKQTKKPRLLVTAAVAAGISNIQAGYEIPQLSQYLDFIHVMTYD
FHGSWEGYTGENSPLYKYPTDTGSNAYLNVEYAMHYWKSNGAPAEKLIVGFPAYGHNFILRDAS
NNGIGAPTSGAGPAGPYTREAGFWAYYEICTFLKEGATVAWDDSQNVPYAYKGTEWVGYDNVNS
FRIKAQWLKENKFGGAMVWAIDLDDFTGTFCNQGKFPLINTLKDSLGLKSAACNASTQPSEPNS
SPGNGSGSGNESSSSESRGYCAGKADGLYPMADNRNAFWNCANGITYEQNCPTGLVFDTSCHCC
NWA
```

Sheep

```
YQLVCYFSNWAQYRPGLGSFKPDNIDPCLCTHLIYAFAGMSNSEITTIEWNDVALYSSFNDLKK
MNSQLKILLAIGGWNFGTAPFTAMVATPENRKTFISSVIKFLHQYGFDGLDFDWEYPGSRGSPS
QDKHLFTVLVQETREAFEQEAKQTKKPRLLVTAAVAAGISNIQAGYEIPQLSQYLDFIHVMTYD
FHGSWEGYTGENSPLYKYPTDTGSNAYLNVEYAMHYWKSNGAPVEKLIVGFPAYGHNFILRDAS
NNGIGAPTSGAGPAGPYTREAGFWAYYEICTFLREGATAGWDDSQNVPYAYKGTEWVGYDNVNS
FRIKAQWLKENKFGGAMVWAIDLDDFTGTFCNQGKFPLINTLKDALGLKSAACNASTQPSEPNS
SPGNGSGSGNESSSSESRGYCAGKADGLYPVADNRNAFWNCANGITYEQNCPPGLVFDTSCHCC
NWA
```

Goat_H128R

```
YQLVCYFSNWAQYRPGLGSFKPDNIDPCLCTHLIYAFAGMSNSEITTIEWNDVALYSSFNDLKK
KNSQLKILLAIGGWNFGTAPFTAMVATPENRKTFISSVIKFLRQYGFDGLDFDWEYPGSRGSPS
QDKHLFTVLVQETREAFEQEAKQTKKPRLLVTAAVAAGISNIQAGYEIPQLSQYLDFIHVMTYD
FHGSWEGYTGENSPLYKYPTDTGSNAYLNVEYAMHYWKSNGAPAEKLIVGFPAYGHNFILRDAS
NNGIGAPTSGAGPAGPYTREAGFWAYYEICTFLKEGATVAWDDSQNVPYAYKGTEWVGYDNVNS
FRIKAQWLKENKFGGAMVWAIDLDDFTGTFCNQGKFPLINTLKDSLGLKSAACNASTQPSEPNS
SPGNGSGSGNESSSSESRGYCAGKADGLYPMADNRNAFWNCANGITYEQNCPTGLVFDTSCHCC
NWA
```

Sheep_H128R

```
YQLVCYFSNWAQYRPGLGSFKPDNIDPCLCTHLIYAFAGMSNSEITTIEWNDVALYSSFNDLKK
MNSQLKILLAIGGWNFGTAPFTAMVATPENRKTFISSVIKFLRQYGFDGLDFDWEYPGSRGSPS
QDKHLFTVLVQETREAFEQEAKQTKKPRLLVTAAVAAGISNIQAGYEIPQLSQYLDFIHVMTYD
FHGSWEGYTGENSPLYKYPTDTGSNAYLNVEYAMHYWKSNGAPVEKLIVGFPAYGHNFILRDAS
NNGIGAPTSGAGPAGPYTREAGFWAYYEICTFLREGATAGWDDSQNVPYAYKGTEWVGYDNVNS
FRIKAQWLKENKFGGAMVWAIDLDDFTGTFCNQGKFPLINTLKDALGLKSAACNASTQPSEPNS
SPGNGSGSGNESSSSESRGYCAGKADGLYPVADNRNAFWNCANGITYEQNCPPGLVFDTSCHCC
NWA
```

Figure S9. Deduced amino acid sequences of the wild and mutant goat and sheep Chia proteins expressed in *E. coli*, related to Figure 3. The amino acid sequences are color-coded, consistent with Figure 3. Green, herbivorous Chia.

White-tailed-deer
YQLVCYFSNWAQYRPGLGSFKPDNIDPCLCTHLIYAFAGMSNNEITTIEWNDVALYSSFNDLKK
KNSQLKILLAIGGWNFGTAPFTAMVATPENRKTFISSVIQFLRQYGFDGLDFDWEYPGSRGSPS
QDKHLFTVLVQETREAFEQEAKQTNKPRLLVTAAVAAGISNIQAGYEIPQLSQYLDFIHVMTYD
FHGSWEGYTGENSPLYKYPTDTGSNAYLNVEYAMNYWKNNGAPAEKLIVGFPAYGHSFTLRDAS
NNGIGAPTSGAGPAGPYTREAGFWAYYEICTFLKDGATEVWDDSQDVPYAYKGTEWVGYDNVNS
FRIKAQWLKENNFGGAMVWAIDLDDFTGTFCNQGKFPLINTLKDALGLKSATCSASTPTSEPSS
NPGNESGSGNESSSSEGRGYCADKADGLYPVANNRNAFWNCANGITYEQNCPTGLVFDTSCHCC
NWA

Musk-deer
YQLVCYFSNWAQYRPGLGSFKSDNIDPFLCTHLIYAFAGMSNNEITTIEWNDVDLYSSFNDLKK
KNSQLKILLAIGGWNFGIAPFTAMVATPENRKTFISSVIKFLRQYGFDGLDYDWEYPGSRGSPS
QDKHLFTVLVQETREAFEQEAKQTNKPRLLVTAAVAAGLSNIQAGYEIPQLSQYLDFIHVMTYD
FHGSWEGYTGENSPLYKSPTDTGSNAYLNVEYAMNYWKNNGAPVEKLIVGFPAYGHNFILRDAS
NNGIGAPTSGTGPAGPYTREAGFWAYYEICTFLKDGATEVWDDSQAVPYAYKGTEWIGYDNVNS
FRIKAQWLKKNNFGGAMVWAIDLDDFTGTFCNQGKFPLINTLKDALGLKSATCDTSTQTSEPNS
SPGTESGTGNEGSISEGRGFCAGKADGLYPVANNRNAFWNCANGITYEQNCPTGLVFDTSCHCC
NWA

Mouse-deer
YQLVCYFSNWAQYRPGLGSFKPEDIDPFLCTHLIYAFAGMSNNEITTIEWNDVTLYKSFNDLKN
KNSQLKTLLAIGGWNFGTAPFTAMVATPDNRKTFISSVVTFLRRYGFDGLDFDWEYPGSRGSPS
QDKHLFTVLVQETREAFEQEAEQTKKPRLLVTAAVAAGISNIKAGYEIPQLSQYLDFIHVMTYD
FHGSQDGYTGENSPLYKYPTDSDDYLNVNSAMNYWKENGAPAEKLIVGFPAYGHNFILSDPSNN
GIGAPTSGAGPAGPYTRQAGFWAYYEICTFLKDGATQAWDDPQDVPYAYKGTEWVGYDNVKSFN
IKAEWLKKYSFGGAMVWAIDLDDFTGTFCSQGKFPLINTLKDALGQKNASYTTSTPTSKPNSST
GSGSDSGSESGSESSSSGSSSGDSGFCAGKADGLYPVANDRNAFWNCANGITYQQNCAAGLVFD
TSCHCCNWA

Giraffe
YQLVCYFSNWAQSRPGLGSFKPDNINPCLCTHLIYAFAGMSNNEITTIERNDVAHYNSFNDLKK
KNSQLKILLAIGGWNFGTALFTAMVATPENRKTFISSVIKFLHQYGFDGLDFDWEYPGSRGSPS
QDKHLFTVLVQETREAFEQEAKQTNKPRLLVTAAVPAGIANIQAGYEIPQLSQYLDFIHVMTYD
FHGSWEGYTSENSPLYKYPTDTGSNAYLNVEYAMNYWKNNGAPAEKLIVGFPAYGHNFVLRDVS
NNGIGAPTSGAGPAGPYTREAGFWAYYEICTFLKDGATEAWDNSQNVPYAYKGTEWVGYDNVNS
FRIKAQWLKENNFGGAMVWAIDLDDFTGNFCNQGKFPLINTLKDALGLKSASCNASTETSEPNS
SPGNESGSGNERSSPEGRGYCAGKADGLYPVENNRNAFWNCANGITYEQNCPTGLVFDTSCHCC
NWA

Figure S10. Deduced amino acid sequences of the herbivorous Chia proteins expressed in *E. coli*, related to Figure 3. The amino acid sequences are color-coded, consistent with Figure 3. Green, herbivorous Chia.

Sperm whale
YQLICYFTNWSQYRPGLGSFKPDNIDPCLCTHLIYAFAGMRNNKITTIEWNDVTLYQSFNSLKN
KNSQLKTLLSIGGWNFGTAPFTAMVSTPENRKTFISSVIKFLRQYGFDGLDLDWEYPGSRGSPS
QDKHLFTVLVQEMREAFEQEAIQTNKPRLLVTAAVAAGISNIQSGYEIPQLSQYLDYIHVMTYD
LHGSWEGYTGENSPLYKYPTDTGNNAYLNVDFAMNYWKNNGAPAEKLIVGFPAYGHNYILSNPS
NNGIGAPTSGAGPAGPYTGQAGFLAYYEICTFLKDGATQAWDAPQYVPYAYKGNEWVGYDNVKS
FHIKAEWLKQNNFGGAMVWAIDLDDFTGTFCNQGKFPLINTLKDVLGLKSASCTTSTQSSEPTR
SAGSGSGSGSGSSSSGGSSVGSGYCAGKANGLYPVANNRNAFLYCSDGITYEQYCQAGLVFDTS
CQCCKLA

Pig
YQLICYFTNWAQYRPGLGSFKPDDIDPCLCTHLVYAFAGMRDNEITTTEGDDVTFYQSFNGLKN
KNSQLKTLLAIGGWNFGTAPFTAMVSAAENRQTFITSVIKFLRQYGFDGLDFDWEYPGSRGSPS
QDKHLFTVLVQEMREAFEQEAKQTKQARLLVTAAVAAGVSNIQSGYEIPQLSQYLDYIHVMTYD
LHASWEGYAGENSPLYKYPTDTGSNAYLNVDYAMNYWKDNGAPAEKLIVGFPAYGHTFLLSNPS
NTDIGAPTSGAGPAGPYTKEAGFWAYYEICTFLKNGATQAWDAPQDVPYAYKGNEWVGYDNVKS
FNIKAQWLKQNNFGGAMVWAIDLDDFTGTFCNQGKFPLINTLKDALGLNSTSCTASAQPSEPSS
GTGSGSTTGSGSGSSSSGSSSGGSGYCAGKADGLYPVANNRNAFWHCQNGITYEQYCQTGLVFD
TSCQCCNWA

Peccary
YQLTCYFTNWAQYRPGVGNFKPDNIDPCLCTHLIYAFAGMSNNEITTIEWDDVTFYKSFNALKN
KNSQLKTLLAIGGWNFGTAPFTTMVSTAENRQTFITSVIKFLRQYGFDGLDLDWEYPGSRGSPS
QDKHLFTVLVQEMREAFEQEAKQTNQPRLLVTAAVAAGISNIQSGYEIPQLSQYLDYIHVMTYD
LHGSWEGYTGENSPLYKYPTDTGSNAYLNVDYAMNYWKDNGAPAEKLIVGFPAYGHTFILSNPS
DTGIGAPTSGAGPAGPYTKQSGFWAYYEICTFLKNGASQAWDGPQDVPYAYKGNEWVGYDDVKS
FSIKAQWLKQNNFGGAMVWAIDLDDFTGTFCNQGKFPLINTLKDALGLKSASCTASAQPSEPSS
GTGSGSGSSSSGSSSGSSGYCAGKANGLYPVANDRNAFWHCVDGITYEQHCQTGLVFDTSCQCC
NWA

Figure S11. Deduced amino acid sequences of the omnivorous Chia proteins expressed in *E. coli*, related to Figure 4. The amino acid sequences are color-coded, consistent with Figure 4. Brown, omnivorous Chia.

(A)

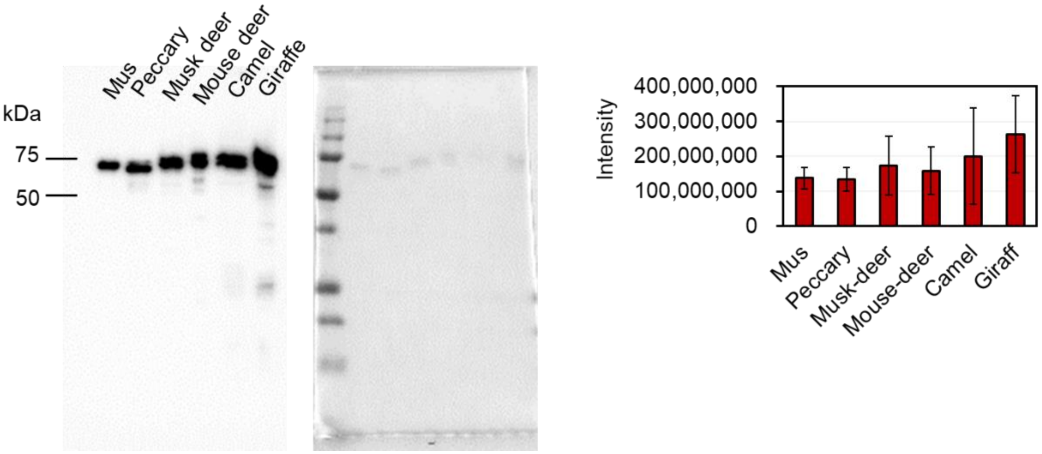

(B)

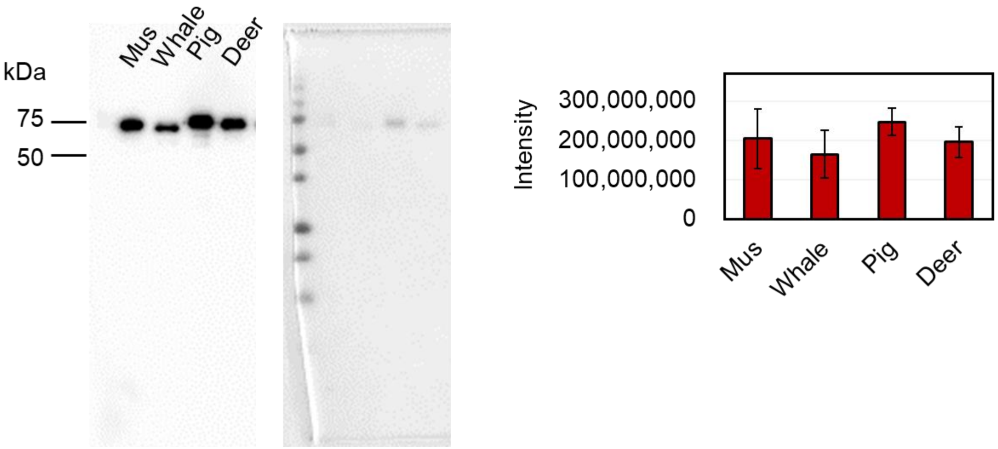

(C)

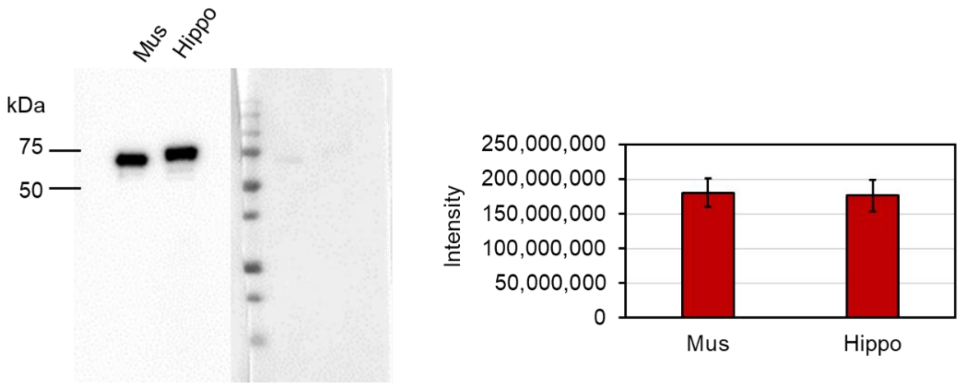

Figure S12. Western blot analysis of the recombinant proteins, related to Figure 4. Each gel image and graph corresponds to the figure in the main text: (A)~(C), Figure 4B. We analyzed the protein fractions using standard SDS-PAGE, followed by Western blot using an anti-V5 antibody. Full-length gel images of Western blots (left panels) and molecular weight markers (middle panels). The signal intensities were quantified in the Star Methods (right panels).

Buffalo Chia5
YQLVCYFSNWAQYRPGLGSFKPDNIDPCLCTHLIYAFAGMSNSEITTIEWNDVALYSSFNDLKK
KNSQLKILLAIGGWNFGTAPFTAMVATPENRKTFISSVIKFLHQYGFDGLDFDWEYPGSRGSPS
QDKHLFTVLVQETREAFEQEAEQTKKPRLLVTAAVAAGISNIQAGYEIPQLSQYLDFIHVMTYD
FHGSWEGYTGENSPLYKYPTDTGSNTYLNVEYAMNYWKNNGAPAEKLIVGFPAYGHNFILRDAS
NNGIGAPISGAGPAGPYTREAGFWAYYEICTFLKDGATEAWDDSQNVPYAYKGTEWVGYDNVNS
FRIKAQWLKENNFGGAMVWAIDLDDFTGTFCNQGKFPLINTLKDALGLKNATCNASTQSSEPNS
SPGKESGSGNKSSSSEGRGYCAGKADGLYPVADNRNAFWNCVNGITYKQNCPTGLVFDTSCHCC
NWA

Buffalo Chia2
YKLVCYLTNWSQYRPEPAKFFPKDMDPCLCTHLIYAFATMNDNKIAPYEWNDIDVLYPEFQALK
EHHKDLVTLLAVGGWNFGTQKFTTMVSSAANRKIFICSIIDFLRQHEFDGIDLDIEYPGSRGSP
PEDKQRFTILIKEMIQAFEEEAEETGKPRLLITAAVSAGKETTDAGYEIAEIGKLLDFISVMTY
DFHGGWDPCTGHNSPLHVGSKDQGNMHYFNCEYAMKYWRDNGVPSEKLIMGFPTYGRTFRLSTS
DTSVCAPVSGAGSSGPYTCEAGFWAYYEICTFLSEATNAWIEDQKVPYAYKDTEWVGYDNIRSY
KYKVDFLKENNFGGAMVWAMDLDDFLGSFCKEGKYPLIRELQSLLGLSSAGSLLSSPHCSKDDA
GQAARMTTAEGQECMRQPQSI

Goat Chia2
YKLVCYFTNWSQYRPEPAKFFPKDVDPCLCTHLIYAFATMNDNKIAPYEWNDIDVLYPEFQALK
EHNKDLVTLLAVGGWNFGTQKFTTMVSSAANRKIFICSVIDFLRQHEFDGIDLDIEYPGSSGSP
AEDKQRFTILIKEMIQAFEEEAEETGKPRLLITAAVSAGKETIDTGYEIAEVGKLLDFISVMTY
DFHGGWDPCTGHNSPLHVGSKDQGNMRYFNCEYAMKYWRDNGVPSEKLIMGFPTYGRTFRLSTS
DTSVCAPVSGAGSSGPYTLEAGFWAYYEICTFLSEATNGWIEDQKVPYAYKDTEWVSYDNIKSC
KCKVDFLKENNFGGAMVWAIDLDDFLGSFCNEGKYPLIHKLQSLLGLSSECTPSATKTWYHQQV
IPRSEGFCANIGGDSGSGGDGGSEGDDGFCTGKADGIYSDPEDNTKFYQCTGGRTFYFQCNQGL
VFDQTCTCCNWPSI

Pig Chia2
YKLVCYFTNWSQYRPDPAKFFPKDVDPCLCTHLIYAFATMNDNKIAPYEWNDIDVLYPQFLALK
ERNKDLVNLLAIGGWNFGTQKFTTMVSTAANRKIFICSVIDFLRQHGFDGIDLDFEYPGSRGSP
PEDKQRFTILIKEMLTAFDEEAKNTGRPRLLITAAVSAGKGTIDAGYEIAEIGKLLDFISVMTY
DFHGGWDTCTGHNSPLHVGSKDEGDMRYFNCEFAMKYWRANGVPSEKLLMGFPTYGRTFRLSTS
DTSVCAPVSGAGSSGPYTREAGFWASYEVCTFLNGATKVWIEDQKVPYAYKDTEWVGYDNIESY
GYKVDFLKENNLGGAMVWAIDLDDFSGSFCNEGKYPLISKLKSLLGLSSECTPPATKTWYNQLV
TPRSEGFCADVGGDGGSGGESGGDGGSGGGGEGGGDGGSGGDDGFCTGKADGIYSDPKDSTKY
YQCAGGRTFHFQCAQGLVFDENCKCCNWPSI

Figure S13. Deduced amino acid sequences of the Chia paralogues expressed in *E. coli*, related to Figure 5. The amino acid sequences are color-coded, consistent with Figure 5. Green, herbivorous Chia; brown, omnivorous Chia.

(A)

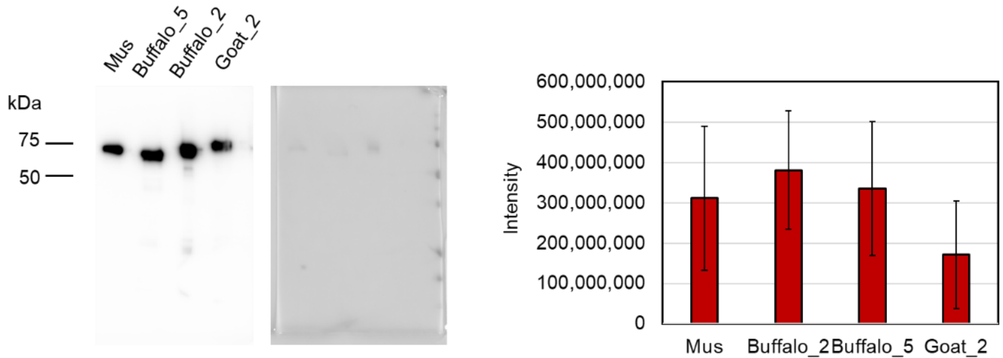

(B)

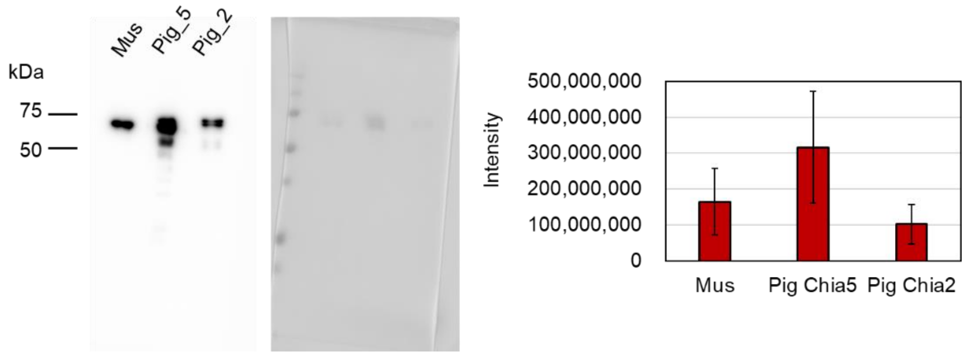

Figure S14. Western blot analysis of the recombinant proteins, related to Figure 5. Each gel image and graph corresponds to the figure in the main text: (A) and (B), Figure 5C. We analyzed the protein fractions using standard SDS-PAGE, followed by Western blot using an anti-V5 antibody. Full-length gel images of Western blots (left panels) and molecular weight markers (middle panels). The signal intensities were quantified in the Star Methods (right panels).

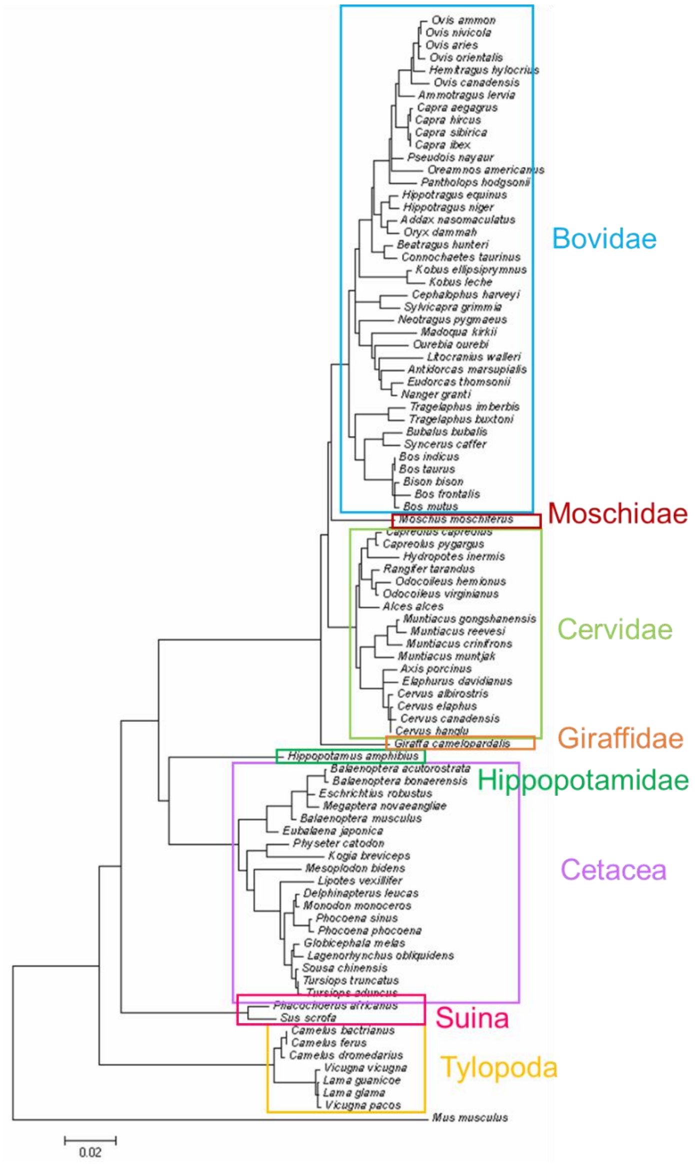

Figure S15. Phylogenetic tree of *Chia,* related to STAR Methods. The evolutionary history was inferred using the Maximum Likelihood method and Kimura 2-parameter model [S1]. A discrete Gamma distribution was used to model evolutionary rate differences among sites (3 categories (+G, parameter = 1.1217)). This analysis involved 89 nucleotide sequences. Evolutionary analyses were conducted in MEGA X [S2].

References

1. Kimura, M. (1980) A simple method for estimating evolutionary rates of base substitutions through comparative studies of nucleotide sequences. J Mol Evol *16*, 111-120
2. Kumar, S., Stecher, G., Li, M., Knyaz, C., and Tamura, K. (2018). MEGA X: Molecular evolutionary genetics analysis across computing platforms. Mol Biol Evol *35*, 1547-1549.
